# Aerobic Exercise Attenuates Autophagy‐Lysosomal Flux Deficits via β2‐AR‐Mediated ESCRT‐III Subunit CHMP4B in Mice With Human MAPT P301L

**DOI:** 10.1111/acel.70184

**Published:** 2025-07-26

**Authors:** Shu‐Guang Bi, Haitao Yu, Tian‐Long Gao, Jia‐Jun Wu, Yu‐Ming Mao, Juan Gong, Fang‐Zhou Wang, Liu Yang, Jia Chen, Zi‐Chong Lan, Meng‐Ting Shen, Yun‐Juan Nie, Gao‐Shang Chai

**Affiliations:** ^1^ Department of Fundamental Medicine, MOE Medical Basic Research Innovation Center for Gut Microbiota and Chronic Diseases, Wuxi School of Medicine Jiangnan University Wuxi Jiangsu China; ^2^ Department of Electrophysiology, Wuhan Children's Hospital (Wuhan Maternal and Children's Healthcare Center), Tongji Medical College Huazhong University of Science and Technology Wuhan Hubei China; ^3^ Department of Pathology Affiliated Hospital of Jiangnan University Wuxi China

**Keywords:** aerobic exercise, Alzheimer disease, autophagy, CHMP4B, MAPT, β2‐adrenergic receptor

## Abstract

Deficits in the autophagy‐lysosomal pathway facilitate intracellular microtubule associated protein tau (MAPT) accumulation in Alzheimer disease (AD). Aerobic exercise (AE) has been recommended as a way to delay and treat AD, but the exact effects and mechanisms have not been fully elucidated. Here, we found that AE (8‐week treadmill running, 40 min/day, 5 days/week) alleviated autophagy‐lysosomal defects and MAPT pathology through the activation of β2‐adrenergic receptors (β2‐AR) in MAPT P301L mice. Molecular mechanistic investigations revealed that endosomal sorting complex required for transport (ESCRT) III subunit charged multivesicular body protein 4B (CHMP4B), which is essential for autophagosome‐lysosome fusion, was significantly decreased in the cerebral cortex of AD patients and the hippocampus of MAPT P301L mice. AE restored the levels of CHMP4B, which reversed autophagy‐lysosomal defects and reduced MAPT aggregation. Inhibition of β2‐AR by propranolol (30 mg/kg, intragastric administration 1 h before each AE session) restrained AE‐attenuated MAPT accumulation by inhibiting autophagy‐lysosomal flux in MAPT P301L mice. Our findings suggest that AE can alleviate autophagosome‐lysosome fusion deficits by promoting the β2‐AR‐RXRα‐CHMP4B‐ESCRT–III pathway, reducing pathological MAPT aggregation, which also reveals a novel theoretical basis for AE attenuating AD progression.

## Graphical abstract

MAPT accumulation inhibited CHMP4B expression and thus disrupted ESCRT‐III‐mediated autophagosome‐lysosome fusion, which induced a vicious cycle between MAPT aggregation and autophagic deficits. Aerobic exercise could effectively block the vicious cycle and reverse autophagic deficits by promoting the β2‐AR‐RXRα‐CHMP4B‐ESCRT‐III pathway, reducing pathologic MAPT aggregation.

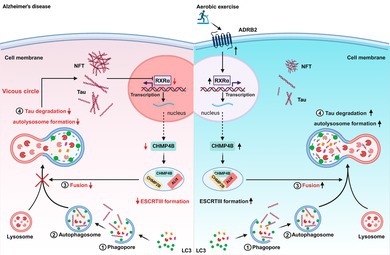

**Abbreviations**
AD: Alzheimer disease
AE: aerobic exercise
ALIX: programmed cell death 6 interacting protein
Aβ: amyloid‐beta
Baf‐A1: Bafilomycin A1
CHMP: charged multivesicular body protein
ESCRT: endosomal sorting complex required for transport
MAPT: microtubule associated protein tau
Prop: propranolol
RXRα: retinoid X receptor alpha
SQSTM1: sequestosome1
TEM: transmission electron microscopy
β2‐AR: β2‐adrenergic receptor

## 1 Introduction

Alzheimer disease (AD) is the most common form of dementia, and the pathological hallmarks of AD include the extracellular accumulation of amyloid‐β (Aβ), the formation of senile plaques (SPs), the intracellular aggregation of hyperphosphorylated microtubule‐associated protein tau (MAPT), the formation of neurofibrillary tangles, synaptic degeneration, and neuronal loss (Hardy and Selkoe 2002; Mawuenyega et al. 2010). In this study, we employed MAPT P301L transgenic PR5 mice, which express human Tau protein carrying the P301L mutation, as a well‐established model of AD (Götz et al. 2001). This model recapitulates key pathological features of tauopathy, including hyperphosphorylated Tau aggregation and neurofibrillary tangle (NFT) formation, and is widely used in mechanistic and therapeutic studies of AD (Santacruz et al. 2005). Studies suggest that autophagic deficits contribute to the accumulation of Aβ and aggregation of MAPT, and upregulation of autophagy has been shown to reduce both Aβ and MAPT levels in the brain (Di Meco et al. 2020; Boland et al. 2008). Several studies have also implicated MAPT in autophagic deficits in AD neurons. For instance, hyperphosphorylated MAPT co‐localizes with LC3‐positive vesicles and autophagic cargo‐receptor SQSTM1/p62, blocking the autophagic process in neurons, evidenced by massive accumulation of autophagosomes in the AD brain (Lee et al. 2001; Piras et al. 2016). More interestingly, recent studies have indicated that MAPT aggregation can repress autophagic flux, and that autophagic flux deficits also can contribute to MAPT aggregation and toxicity in AD (Feng et al. 2020). However, the mechanisms underlying the vicious cycle of MAPT aggregation and autophagic deficits in AD are unclear.

Autophagy is a principal pathway for the degradation of cytoplasmic contents, including damaged or obsolete organelles and abnormal protein aggregates. The complete autophagic process consists of four main consecutive steps: induction, autophagosome formation, autophagosome–lysosome fusion, and degradation (Mizushima et al. 2008; Nixon 2013). This process is generally induced by nutritional deprivation under physiological and pathological conditions, cell stress from damaged organelles, and abnormal/misfolded protein accumulation (Mizushima et al. 2008; Boland et al. 2018). The autophagy‐induced elongating double membrane envelops the targeted substrates via an adaptor protein and then closes to form an autophagosome. Autophagosomes containing targeted substrates are delivered to lysosomes for fusion and degradation through Endosomal Sorting Complex Required for Transport III (ESCRT‐III) (Gatta and Carlton 2019). ESCRT‐III dysfunction induces the conglomeration of autophagosomes and neurodegeneration, which are similar to those observed in the AD‐affected brain (Lee et al. 2007; Rusten et al. 2007). Importantly, increasing evidence indicates that autophagy–lysosomal fusion deficits in neurons are critical preludes to AD, as shown by the substantial accumulation of autophagosomes and autolysosomes in dystrophic neuritis (Feng et al. 2020; Nixon et al. 2005; Moreira et al. 2010; Lauritzen et al. 2016; LaFerla et al. 2007). Therefore, clarifying the role and regulatory mechanism of ESCRT‐III in AD‐related autophagy disorders will help promote the clearance of pathological proteins and alleviate the progression of AD.

An increasing number of studies have revealed that aerobic exercise (AE) or physical activity can be used as a preventive and adjuvant treatment method to retard memory decline in individuals with potential AD as well as in affected patients (Du et al. 2018; Tarumi et al. 2019; Wu et al. 2024; Pitkälä et al. 2013), but the regulatory mechanism and pathway in neurons remain unclear. In the present study, MAPT accumulation inhibited autophagy–lysosomal fusion via the ESCRT‐III subunit CHMP4B. AE reversed autophagy–lysosomal fusion deficits and attenuated MAPT accumulation through the activation of β2‐AR. AE inhibited CHMP4B transcription via its transcription factor retinoid X receptor alpha (RXRα). Our findings suggested that MAPT accumulation inhibited CHMP4B expression and thus disrupted ESCRT‐III‐mediated autophagosome‐lysosome fusion, which induced a vicious cycle between MAPT aggregation and autophagic deficits. AE could effectively block the vicious cycle and reverse autophagic deficits by promoting the β2‐AR‐RXRα‐CHMP4B‐ESCRT‐III pathway, reducing pathologic MAPT aggregation, which also reveals a novel theoretical basis for AE retarding AD progression.

## 2 Results

### 2.1 AE Ameliorates Cognitive Deficits and MAPT Pathology in MAPT P301L Mice

**FIGURE 1:**
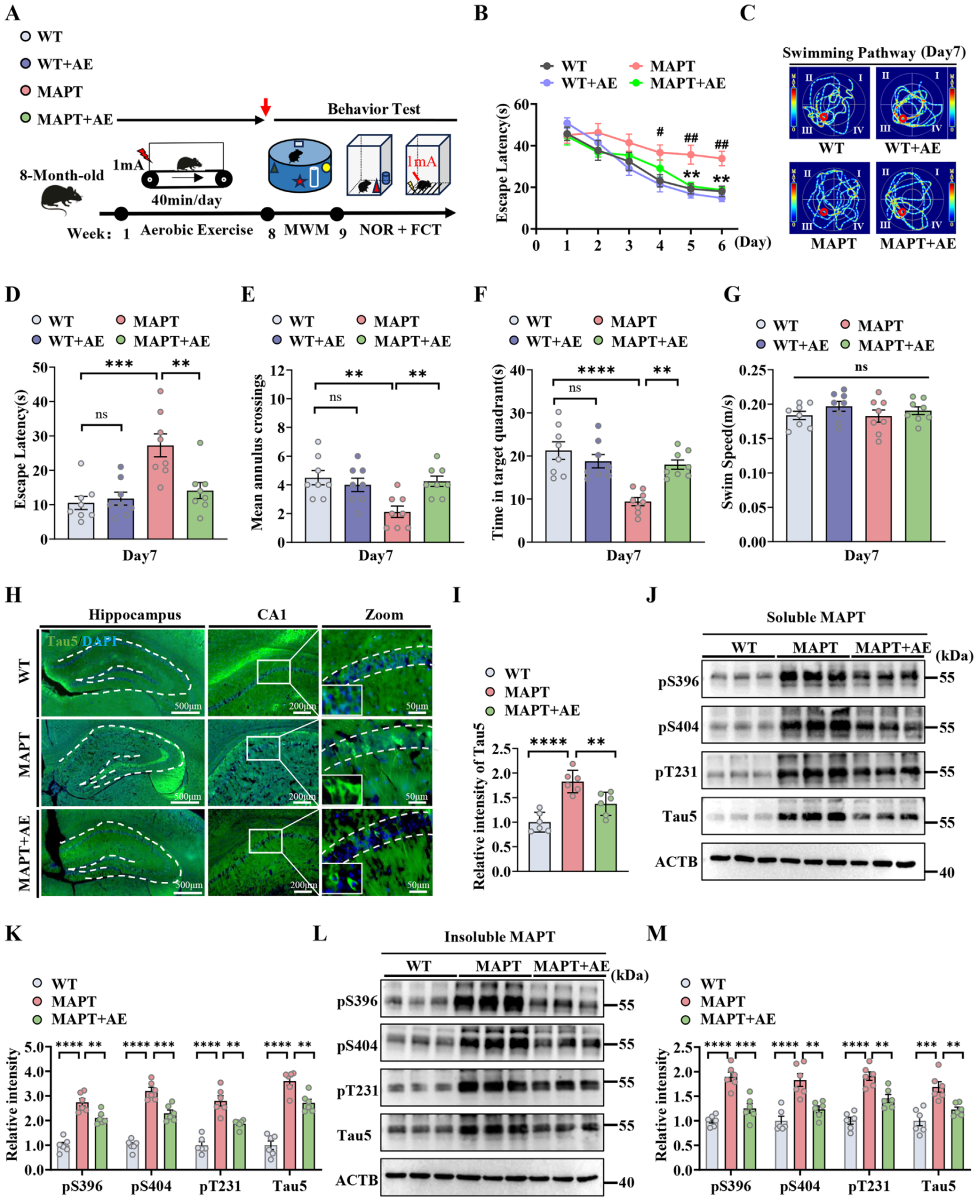
AE ameliorates cognitive deficits and MAPT pathology in MAPT P301L mice. (A) Diagram of the experimental protocol for AE and behavioral tests of WT, WT + AE, MAPT and MAPT+AE mice. ^#^ *p* < 0.05, ^##^ *p* < 0.01 (MAPT vs. WT); *p < 0.05, **p < 0.01, ***p < 0.001 (MAPT+AE vs. MAPT). (B) Escape latency to the hidden platform during the 6‐day training trials. (C) The swimming pathway traveled to locate the platform on day 7. (D) Escape latency, (E) number of crossings of the hidden platform area, (F) the time in target quadrants and (G) swimming speed on day 7. (H and I) Representative images of the fluorescence intensity of Tau5 in CA1 region of the hippocampus (*n* = 6 mice per group). (J–M) The levels of soluble and insoluble Tau of (pS396, pS404, pT231 and Tau5) in the hippocampus were detected using western blotting and quantitatively analyzed (*n* = 6 mice per group). *p < 0.05; **p < 0.01; ***p < 0.001; ****p < 0.0001. The data were presented as the means ± SEMs. Two‐way ANOVA with Bonferroni post hoc test was used for (B), and one‐way ANOVA with Bonferroni post hoc test for the other data.

After 2 months of AE (8‐week treadmill running, 40 min/day, 5 days/week), mice were subjected to behavioral tests for analysis of cognition (Figure 1A). We observed that the MAPT P301L mice presented learning deficits in the Morris water maze (MWM), as revealed by a longer escape latency compared with that of the wild‐type (WT) mice in the training trials, whereas AE effectively decreased the escape latency of the MAPT P301L mice rather than the WT mice from the 5th to 6th days (Figure 1B). In the probe trial on day 7, improved memory was also reflected by a reduced latency to reach the target quadrant, with multiple target quadrant (platform area) crossings and greater exploratory times in the target quadrant (Figure 1C–F). There was no significant difference in swimming speed among these groups (Figure 1G). Moreover, the amelioration of cognitive deficits was identified by a novel object recognition (NOR) test. We found that the AE‐treated MAPT P301L mice rather than the WT mice showed stronger attention and attentional bias toward novel objects (Figure S1A–C). We also measured hippocampus‐ and amygdala‐associated memory via the contextual fear conditioning test (FCT). We found that AE reversed the freezing response of MAPT P301L mice rather than the WT mice in retrieval tests at 2 h and 24 h (Figure S1D–G). These data suggest that AE can ameliorate cognitive deficits in MAPT P301L mice but not in WT mice. Therefore, we chose the WT, MAPT, MAPT+AE mice for further research.

To explore the mechanism underlying the AE‐induced promotion in cognition in MAPT P301L mice, we analyzed the dendritic morphology and spine density of pyramidal neurons in the hippocampal CA1 and DG regions via Golgi staining (Figure S1H). We found that spine density in CA1 and DG neurons was significantly lower in MAPT P301L mice compared to WT mice, but was significantly increased after AE (Figure S1I). We also determined the effects of AE on the expression of synaptic proteins in the MAPT P301L mice. Western blotting revealed that AE induced the upregulation of N‐methyl‐D‐aspartate receptor 1 GRIN1, GRIN2A, GRIN2B, Synapsin I (SYN1) and PSD95 (Figure S1J,K). We further analyzed the ultrastructures of synapses in the hippocampus of the MAPT P301L mice via transmission electron microscopy (TEM) (Figure S1L). The TEM data revealed that synapse intensity and postsynaptic density (PSD) thickness were significantly greater, while the width of synaptic clefts was decreased in the AE‐treated MAPT P301L mice compared to the control mice (Figure S1M–O). In addition, we investigated the effects of AE on neurons. Nissl staining revealed that AE significantly increased the number of neurons in the hippocampal CA1 region of MAPT P301L mice (Figure S1P,Q). Together, these data indicate that AE can normalize synaptic plasticity and increase neuronal numbers in MAPT P301L mice.

Tau hyperphosphorylation and accumulation are the main pathological features of AD and contribute to neurodegeneration and cognitive deficits. To investigate whether AE has beneficial effects on MAPT pathology in MAPT P301L mice, we immunostained Tau5 in brain sections, and the relative fluorescence intensity was quantified (Figure 1H). We observed that AE effectively decreased MAPT accumulation in the hippocampal CA1 region of the MAPT P301L mice (Figure 1I). We further determined the levels of soluble and insoluble MAPT protein in the hippocampus of the MAPT P301L mice. Western blotting revealed that AE significantly decreased total MAPT (Tau5) levels and soluble and insoluble phosphorylated MAPT at multiple AD‐associated sites (Figure 1J–M). Together, these data indicate that AE can promote synaptic plasticity by increasing soluble and insoluble MAPT clearance.

### 2.2 CHMP4B Deficits Underlie MAPT‐Induced Autophagic Repression by Disrupting ESCRT‐III Assembly

**FIGURE 2:**
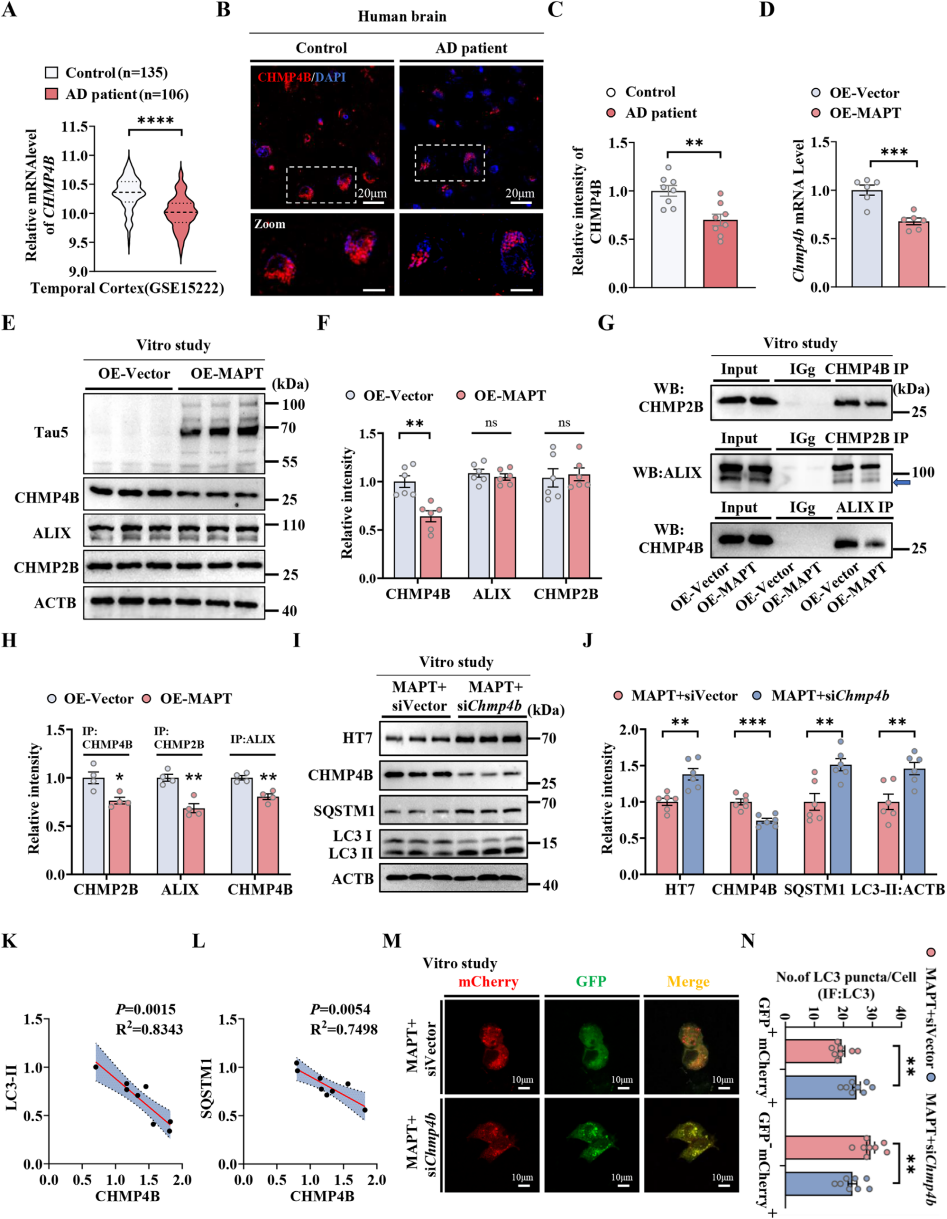
CHMP4B deficits underlie MAPT‐induced autophagic repression by disrupting ESCRT‐III assembly. (A) Compared to the normal control group, CHMP4B was transcriptionally downregulated in the temporal cortex tissue of AD patients in the GSE15222 dataset (control, *n* = 135; AD patient, *n* = 106). (B and C) Representative images and quantification of CHMP4B immunofluorescence intensity in cortical sections from normal controls and AD patients (*n* = 4 per group, 2 sections per individual). (D) The mRNA levels of CHMP4B in N2a cells with overexpression of Vector and MAPT (*n* = 6 independent experiments for each group). (E and F) The levels of Tau5, CHMP4B, ALIX and CHMP2B in N2a cells with overexpression of Vector and MAPT. (G and H) Co‐immunoprecipitation (Co‐IP) assay revealed the interaction among CHMP4B, CHMP2B and ALIX in N2a cells with overexpression of MAPT (*n* = 4 independent experiments for each group). (I and J) The level of HT7, CHMP4B, SQSTM1 and LC3‐II in N2a cells with overexpression of MAPT (*n* = 6 independent experiments for each group). (K and L) CHMP4B protein expression positively correlates with LC3 and SQSTM1 levels in the N2a cells (*n* = 8 independent experiments for each group). (M) Representative images of autophagic flux and (N) quantification of LC3 puncta. (8 images from *n* = 4 independent experiments for each group). *p < 0.05; **p < 0.01; ***p < 0.001; ****p < 0.0001. The data were presented as the means ± SEMs. Unpaired *t*‐test was used to analyze the data.

Autophagic deficits, especially autophagy–lysosomal fusion deficits, play a critical contributing role in pathological MAPT accumulation and clearance impairment (Feng et al. 2020; Nixon et al. 2005; Moreira et al. 2010; Lauritzen et al. 2016; LaFerla et al. 2007). To explore the mechanism underlying autophagic deficits and how AE alleviates tau pathology in the AD brain, we first investigated the key molecules in the AD brain that cause autophagy‐lysosomal fusion deficits. Encouragingly, we found that *CHMP4B* mRNA levels were significantly decreased in the cortical tissues of AD patients, as observed in brain tissue datasets including GSE15222, GSE5281 (temporal cortex), and GSE15222 (frontal cortex) (Figure 2A and Figure S2A,B). Moreover, we performed immunofluorescence staining of CHMP4B in the cortical tissue of the brain from AD patients, and observed the fluorescence intensity of CHMP4B was significantly reduced (Figure 2B,C). CHMP4B is a core member of ESCRT‐III, which is required for the formation of ESCRT‐III and lysosomal degradation of autophagosomes. Therefore, we speculated that CHMP4B may play a role in MAPT‐induced deficits in autophagosome degradation. To confirm the effect of MAPT on CHMP4B, we also determined the mRNA and protein levels of CHMP4B in Neuro‐2a cells (N2a cells, a mouse neuroblastoma cell line) following the overexpression of MAPT and vector. Overexpression of MAPT markedly decreased CHMP4B mRNA and protein levels in N2a cells (Figure 2D–F). CHMP4B interacts with CHMP2B and ALIX, forming a core component of the ESCRT‐III complex (Rusten et al. 2007; McCullough et al. 2008). We first determined the effects of MAPT and CHMP4B on the expression of CHMP2B and ALIX. The knockdown of siChmp4b effects were confirmed by the decreased mRNA and protein levels of CHMP4B in N2a cells (Figure S2C–E). Western blotting revealed that overexpression of MAPT and downregulation of CHMP4B by siRNA had no significant effect on the protein levels of CHMP2B and ALIX (Figure 2E,F and Figure S2D,E). We subsequently determined the effects of MAPT accumulation and CHMP4B reduction on the interactions of CHMP4B, CHMP2B, and ALIX, which play critical roles in the function and assembly of the ESCRT‐III complex and are required for the lysosomal degradation of autophagosomes (Lee et al. 2007). Immunoprecipitation assays revealed MAPT overexpression disrupted the interaction of CHMP4B with CHMP2B or ALIX (Figure 2G,H). In addition, downregulation of CHMP4B decreased the interaction among CHMP4B, CHMP2B, and ALIX (Figure S2F,G). These data indicate that MAPT induces autophagic repression by disrupting CHMP4B‐related ESCRT assembly.

To verify the role of CHMP4B in MAPT‐induced autophagic impairment, we determined the effects of CHMP4B knockdown on autophagy in N2a‐MAPT cells via siRNA. Compared with the control, siRNA targeting *Chmp4b* significantly increased the levels of HT7 (human tau‐specific), LC3‐II, and SQSTM1 in N2a cells, suggesting the accumulation of autophagosomes (Figure 2I,J). Interestingly, a significant correlation between CHMP4B and the LC3‐II and SQSTM1 protein levels was observed (Figure 2K,L). For further analysis of the roles of CHMP4B in autophagic flux, N2a cells were transiently co‐expressed with a tandem mCherry‐GFP‐LC3 fusion protein (Lee et al. 2022) and siRNA‐CHMP4B or si‐Vector, and the numbers of autophagosomes (GFP^+^mCherry^+^, yellow puncta) or mature autolysosomes (GFP^−^mCherry^+^, red puncta) were determined via confocal microscopy (Feng et al. 2020). Compared with those in the si‐Vector‐treated N2a cells, the number of autophagosomes (yellow puncta) was greater and the number of mature autolysosomes (red puncta) was lower in the siRNA‐*Chmp4b*‐treated cells (Figure 2M,N). These data demonstrate that CHMP4B deficits repress autophagy flux via disruption of ESCRT‐III assembly, which may partially be the molecular mechanism underlying autophagy‐lysosomal fusion deficits in AD.

### 2.3 AE Promotes Autophagic Progression by Increasing Autophagosome Degradation in MAPT P301L Mice

**FIGURE 3:**
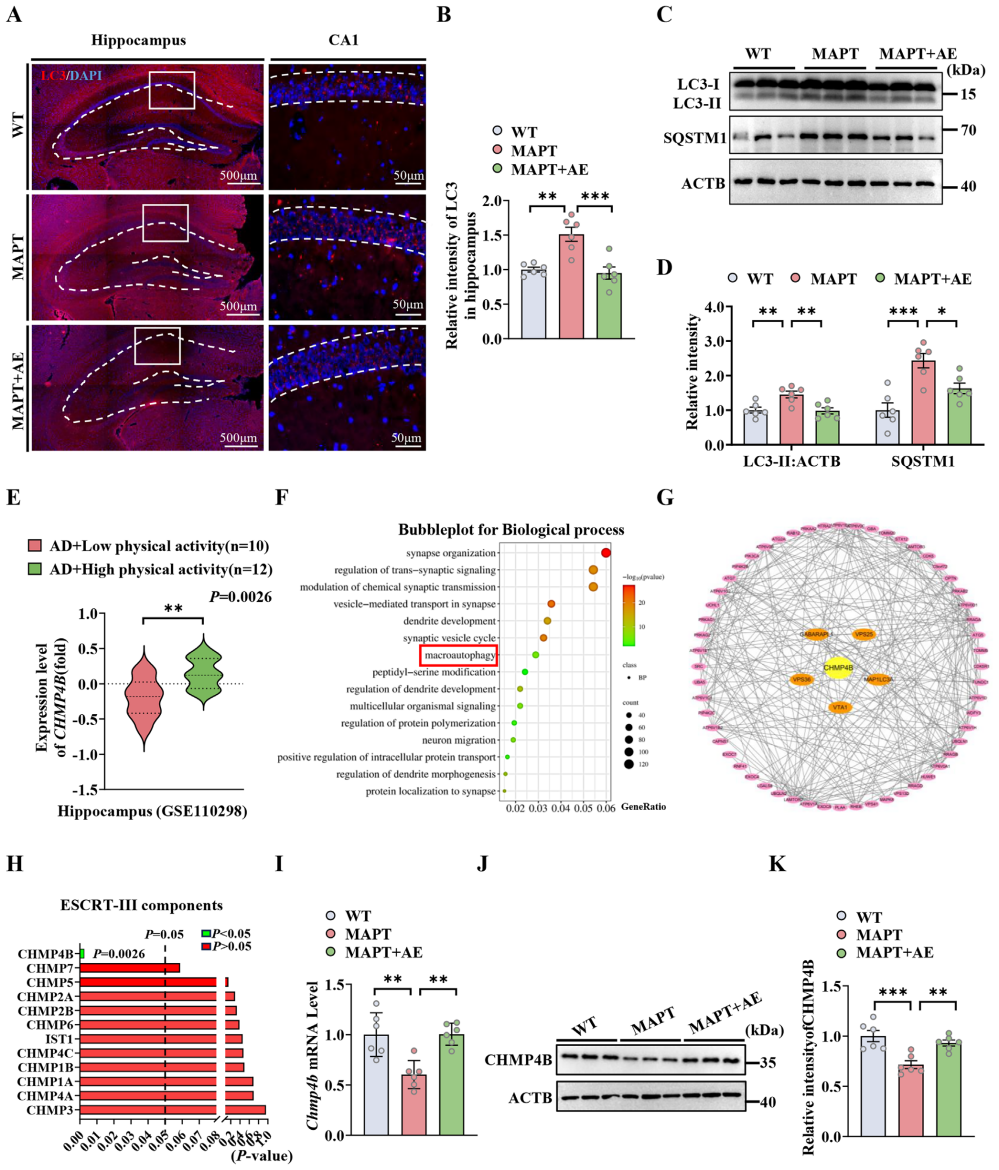
AE promotes autophagic progression by increasing autophagosome degradation in MAPT P301L mice. (A) Representative images of the intensity of LC3 in the hippocampus CA1 region of the mice. (B) Quantification of the LC3 fluorescence intensity in the hippocampus (*n* = 6 mice per group). (C and D) The levels of LC3‐II and SQSTM1 in the hippocampus were detected using western blotting and quantitatively analyzed. (*n* = 6 mice per group). (E) Expression level of DEGs *CHMP4B* in high‐physical‐activity AD patients versus low‐physical‐activity AD patients. (F) Gene ontology (GO) enrichment analysis was performed on upregulation DEGs. (G) Multiple protein interaction analysis revealed that the key Gene *CHMP4B* is associated with other gene networks in macroautophagy. (H) The *p*‐values of ESCRT‐III subunit‐associated genes are shown. Red represents *p*‐values greater than 0.05, while green represents *p*‐values less than 0.05. (I–K) The mRNA and protein levels of CHMP4B in the hippocampus of WT, MAPT and AE‐treated MAPT mice (*n* = 6 mice per group). *p < 0.05; **p < 0.01; ***p < 0.001. The data were presented as the means ± SEMs. Unpaired *t*‐test was used to analyze the data in (E), and one‐way ANOVA with Bonferroni post hoc test for the other data.

To investigate whether AE alleviates MAPT pathology by improving autophagic deficits in MAPT P301L mice, we first measured the effects of AE on LC3‐marked autophagic puncta in the hippocampus of these mice. Immunofluorescence staining showed significantly higher LC3 intensity in MAPT P301L mice compared to WT mice. AE treatment markedly reduced this elevated LC3 immunofluorescence intensity (Figure 3A,B). Upregulation of LC3 indicates increased autophagic induction or accumulation of LC3‐marked autophagosomes (Pyo et al. 2012). We thus examined the levels of autophagy‐associated proteins via western blotting and found that the levels of LC3‐II and sequestosome1 (SQSTM1) in the hippocampal extracts increased, whereas AE reduced these protein levels in the MAPT P301L mice (Figure 3C,D). TEM analysis also revealed that AE decreased the number of autophagic vesicles in the MAPT P301L mice (Figure S3A,B). Immunofluorescence staining revealed that the colabeling of LC3 with Tau5 significantly increased in the MAPT P301L mice. These data indicate that MAPT autophagosome accumulation occurs in the MAPT P301L mice. However, AE attenuated autophagosome accumulation (Figure S3C,D). In addition, the inhibition of autophagy by bafilomycin A1 (Baf‐A1, intraperitoneal [i.p.], 100 nmol/kg) disturbed the AE‐alleviated MAPT pathology in the MAPT P301L mice, as shown by western blotting (Figure S3E,F). Taken together, these findings indicate that AE alleviated MAPT accumulation by promoting autophagic progression in MAPT P301L mice.

To explore how AE normalizes autophagic function, we also assessed the effects of AE on gene expression patterns in the hippocampus of AD patients. In the GSE110298 dataset, high‐physical‐activity AD patients (12 subjects) versus low‐physical‐activity AD patients (10 control subjects) revealed increased transcription of 2895 differentially expressed genes (DEGs), including the autophagy‐related CHMP4B gene (Figure 3E). Gene Ontology (GO) enrichment analysis of upregulated DEGs revealed functions in synapse organization, dendritic development, and macroautophagy (Figure 3F). Moreover, multiple protein interaction analysis revealed that the key CHMP4B gene was associated with other gene molecule networks involved in macroautophagy (Figure 3G). The other subunits of ESCRT‐III were not significantly changed between these two groups (Figure 3H). To confirm the effect of AE on CHMP4B, we determined the mRNA and protein levels of CHMP4B in the hippocampus of MAPT P301L mice; the CHMP4B mRNA and protein levels were substantially reduced in the hippocampus of MAPT P301L mice, whereas AE effectively reversed these changes (Figure 3I–K). Together, these data suggest that AE promotes autophagic progression through the upregulation of CHMP4B, which may be an important mechanism for AE alleviating tau pathology in MAPT P301L mice.

### 2.4 Upregulating CHMP4B Ameliorates Cognitive Deficits and MAPT Pathology by Promoting Autophagosome and Lysosome Fusion in MAPT P301L Mice

**FIGURE 4:**
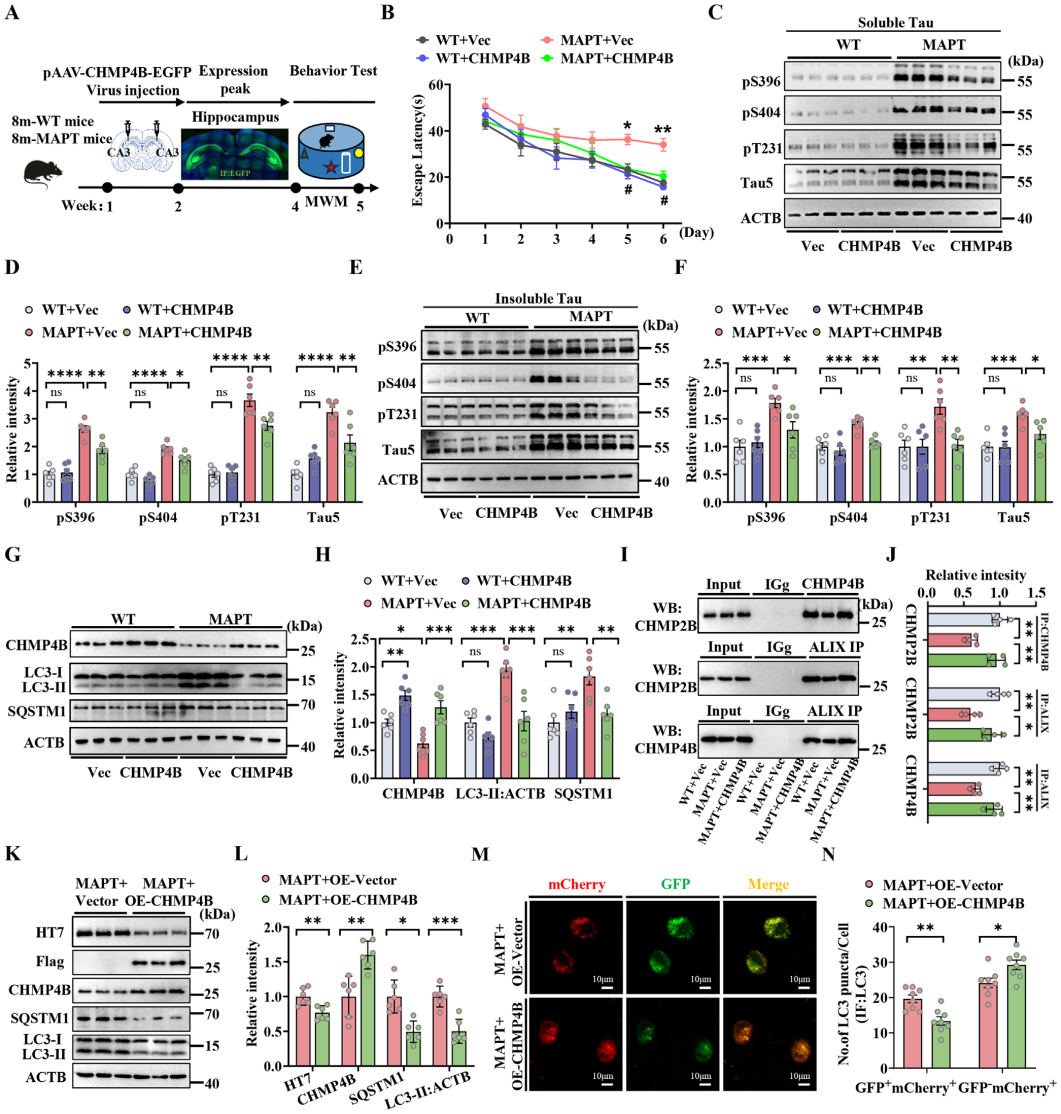
Upregulating CHMP4B ameliorates cognitive deficits and MAPT pathology by promoting autophagosome‐lysosome fusion in MAPT P301L mice. (A) Diagram of the experimental protocol for AAV‐CHMP4B virus injection into the CA3 region of bilateral hippocampus and behavioral tests in WT + Vec, WT + CHMP4B, MAPT+Vec and MAPT+CHMP4B mice. (*n* = 8 mice per group) *p < 0.05, **p < 0.01; (MAPT+Vec vs. WT + Vec); ^#^ *p* < 0.05, ^##^ *p* < 0.01 (MAPT+CHM4B vs. MAPT+Vec); ^*p* < 0.05 (WT + CHM4B vs. WT + Vec). (B) Escape latency to the hidden platform during 6‐day training trials. (C‐F) Levels of soluble and insoluble (Tau of pS396, pS404, pT231 and Tau5) in the hippocampus were detected using western blotting and quantitatively analyzed (*n* = 6 mice per group). (G and H) The levels of CHMP4B, LC3‐II and SQSTM1 in the hippocampus of WT + Vec, WT + AAV‐CHMP4B, MAPT+Vec and MAPT+AAV‐CHMP4B mice (*n* = 6 mice per group). (I and J) Co‐Ip assay revealed the interaction among CHMP4B, CHMP2B and ALIX in the hippocampus of WT + Vec, MAPT+Vec and MAPT+AAV‐CHMP4B mice (*n* = 4 independent experiments for each group). (K and L) The protein levels of HT7, Flag, CHMP4B, LC3‐II and SQSTM1 in N2a cells with overexpression of MAPT and CHMP4B. (M) Representative images of autophagic flux and (N) quantification of LC3 puncta. (8 images from *n* = 4 independent experiments for each group). *p < 0.05; **p < 0.01; ***p < 0.001; ****p < 0.0001. The data were presented as the means ± SEMs. Two‐way ANOVA with Bonferroni post hoc test was used for (B), unpaired *t*‐test was used to analyze the data in (K, N) and one‐way ANOVA with Bonferroni post hoc test for the other data.

To further clarify the key role of CHMP4B in AD autophagy deficiency and determine whether upregulating CHMP4B could ameliorate cognitive deficits in MAPT P301L mice, we constructed an adeno‐associated virus (AAV) expressing CHMP4B (pAAV‐CMV‐CHMP4B‐3Xflag‐EF1a‐EGFP‐tWPA) and stereotaxically injected AAV‐CHMP4B into the hippocampal CA3 region of 8‐month‐old MAPT P301L mice (Figure 4A). After 4 weeks, the overexpression of CHMP4B in the hippocampal CA3 region was confirmed by EGFP fluorescence imaging and western blotting (Figure 4A,G). In the MWM test, compared with the AAV‐NC group of MAPT P301L mice, the MAPT P301L group presented a greater learning ability, as shown by the decreased latency to find the latent platform (5th–6th) during six consecutive days of training (Figure 4B). The improved memory of the AAV‐CHMP4B‐treated MAPT P301L mice was shown by the decreased latency to find the platform area, increased number of crosses in the platform area, and increased time spent in the platform quadrant after the platform was removed on day 7 (Figure S4A–D). There was no significant difference in swimming speed among these groups (Figure S4E). Moreover, we determined the effects of CHMP4B on synaptic plasticity in MAPT P301L mice. Golgi staining revealed that upregulating CHMP4B ameliorated spine degeneration (Figure S4F,G). To further determine whether CHMP4B upregulation could ameliorate pathological MAPT protein expression in MAPT P301L mice, we measured the levels of soluble and insoluble MAPT proteins. We observed that upregulating CHMP4B could ameliorate total MAPT and soluble phosphorylated MAPT at multiple AD‐related sites (Figure 4C–F). In addition, upregulating CHMP4B significantly reduced the LC3‐II and P62/SQSTM1 levels in the MAPT P301L mice (Figure 4G,H). The upregulation of CHMP4B also increased the interaction among CHMP4B, CHMP2B, and ALIX (Figure 4I,J). TEM revealed that upregulating CHMP4B also reversed autophagosome accumulation in the MAPT P301L mice (Figure S4H,I). To verify whether CHMP4B upregulation can rescue MAPT‐induced autophagic impairment, we constructed a 3xFlag‐CHMP4B plasmid to transfect N2a‐MAPT cells. We found that CHMP4B overexpression markedly suppressed the increase in LC3‐II and SQSTM1 levels in N2a‐MAPT cells (Figure 4K,L). By cotransfecting N2a‐MAPT cells with mCherry‐GFP‐LC3 and 3xFlag‐CHMP4B for 48 h, we found that the number of red LC3 puncta (indicating mature autolysosomes) was significantly greater in the CHMP4B‐upregulated cells than in the vector control cells (Figure 4M,N). These data indicate that upregulating CHMP4B promotes autophagosome‐lysosome fusion and rescues MAPT‐induced autophagy impairment, leading to significant attenuation of MAPT pathology in MAPT P301L mice.

### 2.5 AE Upregulates CHMP4B Expression Through β2‐AR‐RXRα Signaling in MAPT P301L Mice

**FIGURE 5:**
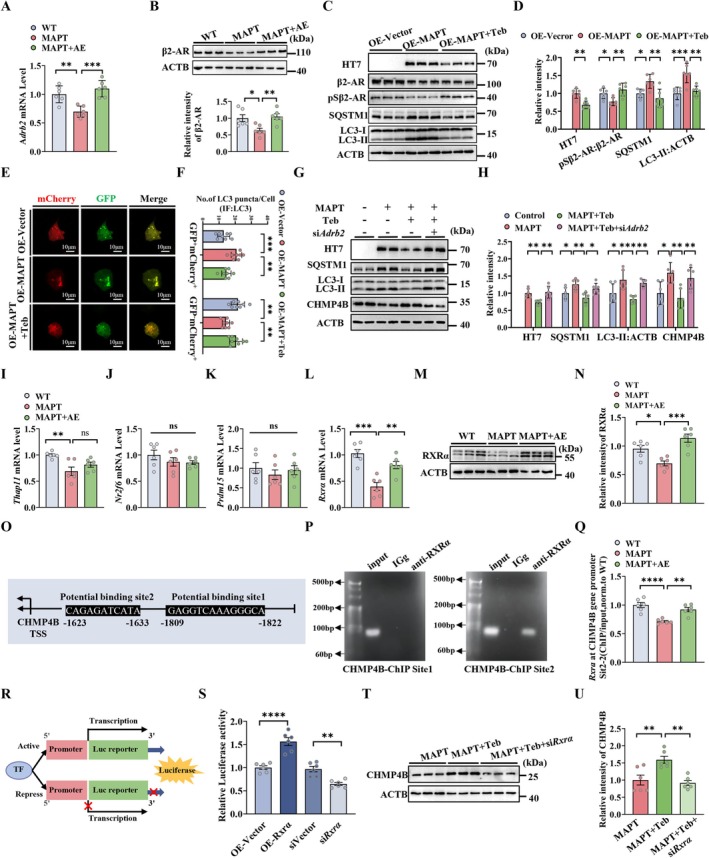
AE upregulates CHMP4B expression through β2‐AR‐RXRα signaling in MAPT P301L mice. (A and B) The mRNA and protein levels of β2‐AR in the hippocampus of WT, MAPT and AE‐treated MAPT mice (*n* = 6 mice per group). (C and D) The level of HT7, β2‐AR, pSβ2‐AR, SQSTM1 and LC3‐II in N2a cells with overexpression of MAPT and treated with Teb (*n* = 6 independent experiments for each group). (E) Representative images of autophagic flux and (F) quantification of LC3 puncta in N2a cells with overexpression of MAPT and treated with Teb (8 images from *n* = 4 independent experiments for each group). (G and H) The protein levels of HT7, CHMP4B, SQSTM1 and LC3‐II in the N2a cells with overexpression of MAPT and CHMP4B and treated with si*Adrb*2 and si*Chmp4b* or Teb detected by western blotting (*n* = 6 independent experiments for each group). (I‐L) The mRNA levels of the *Chmp4b* transcription factors *Thap11*, *Nr2f6*, *Prdm15* and *Rxrα* in the hippocampus of WT, MAPT and MAPT+AE mice (*n* = 6 mice per group). (M and N) The protein levels of RXRα in the hippocampus of WT, MAPT and MAPT+AE mice (*n* = 6 mice per group). (O) Schematic diagram revealing the mouse CHMP4B promoter region and *Rxrα* potential binding Site1 and Site2. (P) ChIP assay results determined the enrichment of RXRα at potential binding site 1 and site 2 of the CHMP4B promoter region. (Q) The direct binding capacity of RXRα at Site2 to the CHMP4B promoter was validated using qPCR of the ChIP products (*n* = 6 mice per group). (R) Diagram of the principle of Dual‐luciferase experiment. (S) Dual‐luciferase reporter gene assays the binding level of RXRα to the CHMP4B promoter at Site2 in N2a cells with overexpression of RXRα and treated with si*Rxrα* (*n* = 6 independent experiments for each group). (T and U) The protein levels of CHMP4B in the N2a cells with overexpression of MAPT and treated with si*Rxrα* or Teb were detected by western blotting (*n* = 6 mice per group). *p < 0.05; **p < 0.01; ***p < 0.001; ****p < 0.0001. The data are presented as the means ± SEMs. One‐way ANOVA with Bonferroni post hoc test was used to analyze the data.

Finally, we aimed to determine how AE rescues MAPT‐induced autophagic repression and how MAPT accumulation suppresses CHMP4B expression. In the central nervous system, β2‐adrenergic signaling is centrally involved in the regulation of neurological function and activity, including cognition and immunity (Xu et al. 2023; Li et al. 2013). More recently, we reported that β2‐adrenergic signaling was upregulated in AE‐treated AD patients and APP/PS1 mice (Wu et al. 2024). Mechanistically, we speculate that β2‐AR is a key receptor that mediates AE to regulate neurological function. Therefore, we measured the activation and expression of β2‐AR in the hippocampus of MAPT P301L mice. RT‐PCR and western blotting revealed that the mRNA and protein levels of β2‐AR were significantly reduced in the hippocampus of the MAPT P301L mice, whereas AE reversed these changes (Figure 5A,B). While AE treatment produced no significant alterations in β1‐AR or β3‐AR expression levels (Figure S5A,B). To determine whether AE normalizes autophagy through β2‐AR signaling in MAPT P301L mice, we treated N2a‐MAPT cells with the β2‐AR‐specific agonist terbutaline (Teb) and measured the levels of β2‐AR, pSβ2‐AR (Ser355/356), LC3 and SQSTM1. We found that the activation of β2‐AR markedly suppressed the MAPT‐induced upregulation of LC3 and SQSTM1 accompanied by a decrease in MAPT in N2a‐MAPT cells (Figure 5C,D). Correspondingly, we also observed that the activation of β2‐AR can attenuate MAPT‐induced deficits in autophagosome and lysosome fusion, as shown by an increased number of mature autolysosomes (red puncta) (Figure 5E,F). In addition, we found that the activation of β2‐AR can upregulate CHMP4B expression, which is accompanied by a decrease in LC3 and SQSTM1, and that si‐*Adrb2* effectively inhibited the above changes induced by Teb treatment (Figure 5G,H). The decrease in *Chmp4b* mRNA in MAPT P301L mice and N2a‐MAPT cells indicated the involvement of transcription. To identify potential transcription factors regulating chmp4b, we retrieved a 2000 bp sequence upstream of its transcription start site from the NCBI database, representing the putative promoter region (Wu et al. 2024; Wang et al. 2024). We then screened for potential binding sites of the *Chmp4b* promoter in the NCBI database and predicted potential transcription factors in the JASPAR database to gain insight into the molecular mechanism of the decrease in CHMP4B (Sandelin et al. 2004). Based on binding affinity scores and integrated analysis, we identified four top‐ranked candidate genes: *Thap11*, *Nr2f6*, *Rxrα and Prdm15* (Figure S5C). Compared with those in WT mice, the mRNA levels of *Thap11* and *Rxrα* were significantly lower in the hippocampus of hMAPT mice and N2a‐MAPT cells (Figure 5I–L and Figure S5D–G). Interestingly, AE effectively upregulated the expression of the transcription factor RXRα at the mRNA and protein levels (Figure 5L–N). We further verified the direct binding site (CHMP4B TSS2‐1809/‐1822, but not CHMP4B TSS1‐1623/‐1633) of RXRα to CHMP4B via a chromatin immunoprecipitation (ChIP) assay and RT–PCR in N2a cells (Figure 5O–Q). In addition, we inserted the CHMP4B‐ChIP sequence into luciferase reporter gene plasmids to validate the regulation of CHMP4B expression by RXRα. A dual‐luciferase reporter gene assay revealed that the binding of RXRα to CHMP4B upregulated the expression of CHMP4B in N2a cells (Figure 5R,S). Activation of β2‐AR can upregulate the expression of CHMP4B, while knocking down RXRα inhibited these effects, as shown by western blotting in N2a‐MAPT cells (Figure 5T,U). These data indicate that MAPT accumulation can decrease CHMP4B expression by inhibiting THAP11 and RXRα. AE and activation of β2‐AR can upregulate RXRα, promoting CHMP4B expression. In turn, elevated CHMP4B may block the vicious cycle of pathological MAPT accumulation and autophagy impairment.

### 2.6 The β2‐AR Inhibitor Propranolol Reverses the Ameliorative Effects on Cognition and Pathology in MAPT P301L Mice After AE

**FIGURE 6:**
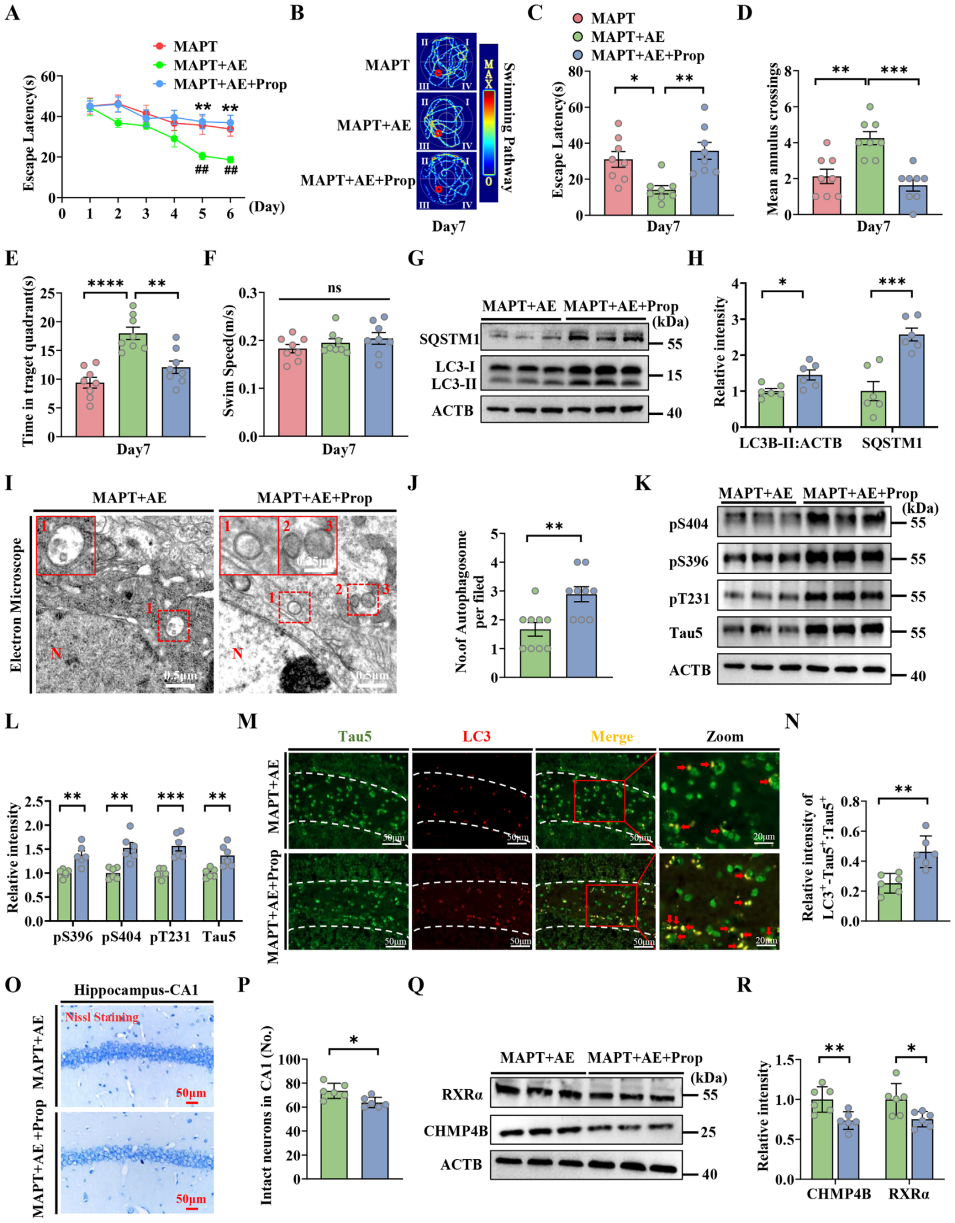
The β2‐AR inhibitor propranolol reverses the ameliorative effects on cognition and pathology in MAPT P301L mice after AE. (A) Escape latency to the hidden platform during the 6‐day training trials in the MWM test (*n* = 8 mice per group). *p < 0.05, (MAPT+AE vs. MAPT); ^#^ *p* < 0.05, ^##^ *p* < 0.01 (MAPT+AE + Prop vs. MAPT+AE). (B) The swimming pathway traveled to locate the platform on day 7. (C) Escape latency, (D) number of crossings of the hidden platform area, (E) the time in target quadrants and (F) swimming speed on day 7. (G and H) The protein levels of SQSTM1 and LC3‐II in the hippocampus of the AE and propranolol‐inhibited groups (*n* = 6 mice per group). (I and J) Electron microscopy analysis of autophagosomes shows arrows indicating autophagosomes (*n* = 9 images from 3 mice per group; scale bar: 0.5 μm). (K and L) The protein levels of soluble Tau of (pS396, pS404, pT231 and Tau5) in the hippocampus were detected using western blotting and quantitatively analyzed. (M) Representative confocal images of LC3 and Tau5 immunofluorescence co‐labeling in the DG region of the hippocampus of the mice. (N) Quantification of the ratio of LC3^+^Tau5^+^:Tau5^+^. (O and P) Nissl staining and quantification of intact neurons in the hippocampal CA1 region of mice. (*n* = 6 mice per group). (Q and R) The protein levels of RXRα and CHMP4B in the hippocampus of the AE and propranolol‐inhibited groups (*n* = 6 mice per group). *p < 0.05; **p < 0.01; ***p < 0.001; ****p < 0.0001. The data are presented as the means ± SEMs. Two‐way ANOVA with Bonferroni post hoc test was used for (A), and one‐way ANOVA with Bonferroni post hoc test was used to analyze the data in (C–F) and unpaired *t*‐test for the other data.

To determine whether the ameliorated cognitive deficits and MAPT pathologies are exerted through β2‐AR, we treated mice with propranolol (β2‐AR inhibitor) before AE. We observed that propranolol interferes with learning and memory in the training (1–6 days) and testing (7th day) phases of the MWM test (Figure 6A–F). We observed that the protein levels of LC3‐II and SQSTM1 were significantly increased after propranolol pretreatment compared with those in the mice treated with AE alone (Figure 6G,H). Moreover, TEM revealed that the number of autophagic vesicles was similarly increased in the propranolol‐pretreated mice (Figure 6I,J). We also determined the effects of propranolol on MAPT pathologies. Consistent with these findings, the total MAPT levels and phosphorylated MAPT significantly increased after propranolol treatment (Figure 6K,L). These data suggest that propranolol can reverse the ameliorative effects on cognition and pathology in MAPT P301L mice after AE. Compared with that in the AE‐treated group, immunofluorescence staining of the colabeling of LC3 with Tau5 significantly increased after propranolol pretreatment. These data indicated that propranolol aggravated autophagosome accumulation (Figure 6M,N). In addition, propranolol treatment exacerbated neuronal loss in MAPT P301L mice, showing reduced NeuN^+^ cells, suggesting β2‐AR‐mediated autophagy‐lysosome flux defect affects neuronal survival (Figure 6O,P). We similarly examined CHMP4B and RXRα levels after treatment with propranolol and found that propranolol treatment significantly inhibited the effect of AE on the increased expression of CHMP4B and RXRα in the MAPT P301L mice (Figure 6Q,R). Together, these results suggest that AE ameliorates autophagic impairment in MAPT P301L mice via β2‐AR‐RXRα‐CHMP4B‐ESCRT‐III pathway signaling.

## 3 Discussion

Many studies on AD have focused on discovering drugs that can restrain Aβ and tau accumulation and/or promote Aβ and tau clearance, including antibodies and kinase inhibition. However, most of these methods fail to retard the progression of cognitive impairment and cause some side effects, including neuroinflammation and other neuropathologies (Doody et al. 2014; Liu et al. 2012). Therefore, especially in the early stages, lifestyle intervention is considered a key strategy for mitigating the progression of AD (Nicola et al. 2024). In recent years, an increasing number of studies have confirmed that AE not only promotes physical function but also has significant preventive or beneficial effects on neurological and psychiatric disorders (Hillman et al. 2008; Hansen et al. 2020; Abdullahi et al. 2024; Pearce et al. 2022). Importantly, evidence from systematic reviews and meta‐analyses has shown beneficial effects of AE on cognitive decline in patients with AD as well as individuals with potential AD (Du et al. 2018; Pitkälä et al. 2013; Brasure et al. 2018; Boa Sorte Silva et al. 2024). Although many mechanisms have been reported to modify the effects of AE in AD, including improved vascular function (Pedrinolla et al. 2020), increased brain energy metabolism (Castellano et al. 2017) and electrical activity (Parvin et al. 2020), and amelioration of inflammation, hyperphosphorylated MAPT and Aβ load (Wu et al. 2024; Jensen et al. 2019; Ohia‐Nwoko et al. 2014), the underlying molecular and cellular mechanisms are still unclear, especially how AE regulates nerve cells to reduce MAPT and Aβ accumulation.

Deficits in the autophagy‐lysosomal pathway have been implicated in the process of neurodegenerative disease (Jegga et al. 2011). However, the molecular mechanism underlying autophagic deficits, especially autophagy–lysosomal fusion dysfunction, in AD remains unclear. The course of fusion involves ESCRTs, the small GTPase RAB7 (Xing et al. 2021), soluble N‐ethylmaleimide‐sensitive factor attachment proteins (SNAREs) (Itakura et al. 2012), and lysosomal‐associated membrane protein 2 (LAMP2) (Qiao et al. 2023), in which the ESCRT machinery functions in sorting transmembrane cargo proteins to lysosomes for degradation (Vietri et al. 2020). ESCRTs are widely expressed in the central nervous system and are involved in neurodegeneration, as shown by the abnormal fusion process of autophagosomes into autolysosomes (Lee et al. 2007; Lee and Gao 2008; Skibinski et al. 2005). The ESCRT machinery is subdivided into 3 functional subcomplexes known as ESCRT‐I, ESCRT‐II, and ESCRT‐III (Katzmann et al. 2001; Schöneberg et al. 2017; Babst et al. 2002). CHMP4B is a core member of ESCRT‐III, which is required for the formation of ESCRT‐III and lysosomal degradation of autophagosomes (Lee et al. 2007; Zhen et al. 2020; Vietri et al. 2015; Jimenez et al. 2014; Radulovic et al. 2018). Here, we observed that MAPT accumulation induced autophagy–lysosomal fusion deficits accompanied by a decrease in CHMP4B expression in the brains of MAPT P301L mice. Further study suggested that MAPT decreased CHMP4B via the inhibition of the transcription factor RXRα and that CHMP4B may be the key to causing autophagy‐lysosomal fusion disorder by inhibiting the formation of ESCRT‐III.

AE has a wide range of beneficial effects on chronic diseases, including neurodegenerative disease, cardiovascular disease, and metabolic syndrome (Handschin and Spiegelman 2008; De Miguel et al. 2021). Recent studies have shown that AE or physical exercise likely modulates the levels of Aβ and tau in both human and rodent models (Wu et al. 2024; Ohia‐Nwoko et al. 2014; Desai et al. 2021; Devanand et al. 2023). However, the molecular mechanisms by which AE elicits benefits are unclear, limiting the potential of developing therapeutic interventions for AD. Our results demonstrated that AE significantly improved cognitive deficits in MAPT‐P301L mice, as evidenced by significant behavioral improvements in the Morris water maze (spatial memory), novel object recognition (recognition memory), and contextual fear conditioning (associative learning). In particular, the effects of AE on pathological MAPT elimination by autophagy are largely unknown. Our study revealed that AE can ameliorate pathological MAPT accumulation mainly by increasing autophagy. While our study primarily focused on the therapeutic role of AE in reversing established Tau pathology, emerging evidence suggests that early AE intervention may also exert preventive effects by promoting autophagy, reducing neuroinflammation, and maintaining metabolic homeostasis. This possibility warrants further investigation in younger animal models prior to the onset of pathological Tau accumulation. To investigate the mechanism by which AE alleviates autophagic deficits, we further combined sequencing analysis of AD patients (GSE110298) and identified CHMP4B, a core subunit of ESCRT‐III that is required for the activation and formation of ESCRT‐III. We observed that the knockdown of CHMP4B impedes the association of CHMP2B with ALIX, which disrupts the assembly of ESCRT‐III and induces autophagy–lysosomal flux deficits, increasing pathological MAPT accumulation in N2a cells. We also found that CHMP4B was significantly reduced in the brains of AD patients, suggesting a novel neurodegenerative mechanism that may have at least partial implications for understanding autophagy–lysosomal fusion deficits in AD. Encouragingly, the upregulation of CHMP4B ameliorated pathological MAPT and cognitive impairments in MAPT P301L mice. Thus, CHMP4B may represent a promising therapeutic target for tauopathies via autophagy in AD.

To clarify how AE regulates CHMP4B expression and reverses MAPT‐induced autophagic deficits, we identified the transcription factor RXRα for AE‐regulated CHMP4B expression. In addition, the regulatory receptors of AE on RXRα were explored in MAPT mice. We recently demonstrated the effectiveness of AE in attenuating Aβ pathology by increasing autophagy in amyloid beta precursor protein (APP)‐PSEN1/PS1 mice (Wu et al. 2024). Further exploration of the underlying mechanism has revealed the potential role of β2‐AR, which plays an important role in the regulation of energy metabolism (Zahalka et al. 2017). β2‐AR, a member of the G‐protein coupled receptor family, is abundantly expressed in neurons and microglia. Previous studies have shown that activation of β2‐AR through an enriched environment can alleviate hippocampal LTP (Long‐term potentiation) impairment and neuroinflammation induced by Aβ in vivo (Xu et al. 2023; Li et al. 2013). Combining the insight into the interplay between AE and β2‐AR, we speculate that AE may regulate CHMP4B through β2‐AR. Therefore, we tested the effects of β2‐AR on CHMP4B expression and observed that the activation of β2‐AR could mimic the AE‐induced upregulation of CHMP4B in N2a‐MAPT cells. Correspondingly, β2‐AR‐specific inhibitors reduced the AE‐induced upregulation of CHMP4B and ameliorated autophagy‐lysosomal flux deficits. Thus, AE via β2‐AR‐CHMP4B‐ESCRT‐III attenuated autophagy–lysosomal flux deficits to eliminate pathological MAPT, which has important implications for AD treatment.

## 4 In Summary

Our findings suggest a neurodegenerative mechanism: pathological MAPT inhibits CHMP4B expression via RXRΑ, and loss of CHMP4B disrupts the activation and formation of ESCRT‐III and obstructs autophagosome‐lysosome fusion, resulting in autophagic deficits (Figure S6). This process mutually aggravates pathological MAPT accumulation and autophagic impairments, resulting in a vicious cycle in the progression of AD. AE can impede the vicious cycle through β2‐AR‐RXRα‐CHMP4B and normalize the autophagy‐lysosome pathway to clear pathological MAPT, which ultimately ameliorates cognitive deficits.

## 5 Materials and Methods

### 5.1 Mice, AE, Drug Distribution, and Brain Stereotaxic Injection

All animal experimentation procedures received approval from the Animal Ethics Committee of Jiangnan University (JN. No20240630t0321231[379]). We used MAPT P301L mice as AD mouse models, transgenic PR5 mice carrying the human Tau P301L mutation (Götz et al. 2001; Götz and Ittner 2008), and age‐matched WT PR5 mice serving as controls. MAPT P301L mice were donated by Prof. Yan‐Jiang Wang from the Third Military Medical University (Mañucat‐Tan et al. 2019). All the mice were 8 months old, with an equal distribution of males and females, and were maintained on a 12‐h light/dark cycle with full access to food and water.

The mice began running on a 6‐channel treadmill (TECHMAN, Chengdu, China) at 8 months of age for 40 min a day, 5 days a week, over a period of 8 weeks (Ohia‐Nwoko et al. 2014). The exercise intensity was set at 50% or 75% of its maximal speed, a range that has been widely recognized as suitable for sustained long‐term aerobic training (Vieira et al. 2007; Li et al. 2024). With a progressive treadmill protocol, the mice with AE were able to reach up to 15 m/min. The protocol was followed with a specific sequence: 5 min at 6 m/min, followed by 5 min at 9 m/min, 20 min at 12 m/min, 5 min at 15 m/min, and 5 min at 12 m/min, all while maintaining a slope of 0°. To control for environmental influences during the training phase, non‐exercised WT and MAPT mice were placed in the exercise facility under conditions identical to those of the AE group. Mice were treated with the systemic β2‐AR antagonist propranolol (MedChemExpress, HY‐B0573B) at a dose of 30 mg/kg by intragastric administration 1 h prior to the AE (Tsai et al. 2020). MAPT mice were treated with Baf‐A1 (100 nmol/kg, i.p.) (MedChemExpress, HY‐100558), an inhibitor of autophagosome and lysosome fusion, or received vehicle injections (5% DMSO in 0.9% NaCl) 1 h prior to the AE (Zapata et al. 2022). For adherence to the exercise protocol, the mice underwent exercise endurance assessments throughout the regimen.

The AAV with CHMP4B (pAAV‐CMV‐CHMP4B‐3Xflag‐EF1a‐EGFP‐tWPA) was produced by OBiO Technology (Shanghai, China). For virus injection, the 8‐month‐old mice were injected with 1.25% tribromoethyl alcohol (1 mL/100 g) by intraperitoneal injection and then stereotaxically injected with 0.5 μL of CHMP4B‐AAV (5.49^12^ TU/ml) into the hippocampal CA3 region (bregma: anterior/posterior −2 mm, medial/lateral ±2.5 mm, and dorsal/ventral −2.3 mm) via a Hamilton microsyringe at an infusion rate of 0.2 μL/min. After injection, the microsyringe needle was left in the brain for 10 min (Zhou et al. 2023). After the injection, the needle was retained in situ for 10 min before the wound was sutured, allowing the mice to recover appropriately.

### 5.2 Human Brain Samples

Frozen autopsy brain samples of human post‐mortem cortical tissue sections from AD patients and healthy controls were obtained from Guizhou Medical University, and Table 3 provides more details. The use of human brain tissue samples was authorized by the ethics committee of Guizhou Medical University and the ethics committee of Jiangnan University (JNU202403RB095).

### 5.3 Tissue Extraction

Following the completion of the behavioral test, the mice were permitted to become familiar with the laboratory environment for 2 h, followed by the administration of anesthesia to the mice via a 20% solution of urethane (10 mL/kg, administered intraperitoneally). During the onset of the effects of urethane, manipulation tools, equipment for perfusing the heart, and an ice tray were all set up. Once the absence of limb tendon reflexes was observed, the limb was secured to a foam board with a needle, the skin of the chest was pulled up with forceps, and with the other hand, scissors were used to cut the skin and ribs of the chest cavity to expose the heart and liver. The injection needle was carefully placed into the left ventricle, which was positioned 1–2 mm to the left of the apex of the heart and aligned with the aortic arch. Subsequently, 100 mL of preheated physiological saline (maintained at 37°C) was perfused over a period of 5 min. Following perfusion, the brain tissue was placed on ice, followed by swift dissection of the right hippocampus and cortex, which were then promptly stored in containers at −80°C. The remaining hemisphere was fixed in a 4% paraformaldehyde solution for further analysis.

### 5.4 Morris Water Maze (MWM)

The MWM test was implemented with a few modifications, as previously described (Vorhees and Williams 2006). The mice were acclimated to the experimental environment for 24 h prior to behavioral testing. A circular pool (120 cm in diameter, 60 cm in height) was filled with water to a depth of 40 cm, which was rendered opaque by adding milk and maintained at 20°C–22°C. The pool was virtually divided into four equal quadrants, each marked with distinct colors and geometric patterns as visual cues. A transparent platform (10 cm × 5 cm) was submerged 1 cm below the water surface in the target quadrant (quadrant III). The mice learned 4 times a day to randomly search for transparent platforms from different quadrants for 60 s during the initial six days of instruction. The mice were led to the platform and given a 30 s grace period if they could not locate the platform within 60 s. On the 7th day, the test was conducted again with the platform absent, and the mice began from the contralateral quadrant of the original platform location. In this experiment, mice were considered to have found the platform if they climbed onto it and remained for more than 1 s. Following testing, the mice were immediately dried with absorbent paper and placed on a dry towel for thermal support. The escape delay, number of platform crossings, and target quadrant dwell duration were determined and assessed via MWM analytical software (WMT‐100S, TECHMAN, China).

### 5.5 Novel Object Recognition (NOR)

The NOR test was performed with minor modifications as described in detail (Brunson et al. 2005). The day before training, mice underwent a 5‐min habituation session in an empty arena (a 60 × 60 × 50 cm3 plastic container). On the first training day, two objects (A and B) were placed in the arena, positioned 3 cm from the inner walls at opposite corners. Each mouse was then placed in the arena facing the wall and allowed 5 min for exploration. After 24 h, object A was exchanged for a novel object C, which was made of the same material and size but had a different shape for the mice to recognize. Between each trial, the arena and objects were thoroughly cleaned with 70% ethanol to minimize olfactory cues. The duration that the mice spent exploring object C was recorded as 5 min, and the ratio of time that the mice spent exploring novel C to that spent exploring the previously performed object was regarded as a measure of recognition memory.

### 5.6 Fear Conditioning Test (FCT)

The day before testing, animals were placed in a fear conditioning chamber (Taimeng FCT100, China) for a 3‐min acclimation period. For the fear conditioning test, there were two stages to the FCT: training and testing. During the training period, the mice were placed in an experimental box with electrical stimulation on the bottom. The mice were subsequently subjected to 28 s of noise, 2 s of 1 mA current stimulation, and a 30 s interval per cycle 3 times. The mice were subsequently tested with only 28 s and no electrical stimulation for the subsequent 2 s at 2 h and 24 h later. The chamber was cleaned with 75% ethanol after each animal to control for odor cues. The freezing time of the mice was automatically determined by analysis software (FCT100, Taimeng, China).

### 5.7 Western Blotting

The brain tissue was homogenized, lysed, and then electrophoresed through polyacrylamide gels (Vazyme Biotech Ltd.) for approximately 2 h, after which the proteins were transferred to PVDF membranes (Millipore, USA). The membranes were subsequently blocked with 5% bovine serum albumin (BSA) solution at room temperature for 1 h and then incubated with primary antibodies overnight. Finally, the blots were analyzed quantitatively via ImageJ after the membranes were incubated with a secondary antibody (anti‐rabbit or anti‐mouse IGg conjugated to horseradish peroxidase, 15,000) solution for 1 h at room temperature. The blots were subjected to quantitative analysis via ImageJ. A comprehensive list of the primary antibodies used is provided in Table 2.

### 5.8 Coimmunoprecipitation

Coimmunoprecipitation was performed with minor adjustments as previously described (Lin and Lai 2017), and the results were measured by using a kit (Beyotime, P2197S). A total of 10 mg of hippocampal tissue was homogenized via radioimmunoprecipitation assay lysis buffer with a cryogenic homogenizer and then centrifuged at 10,000 × g at 4°C for 5 min, after which approximately 100 μL of homogenate was collected from the supernatant. The 20 μL anti‐CHMP4B and A + G agarose gel was washed with PBS 3 times for 30 s and then incubated with 100 μL of primary antibody diluted at room temperature for 1 h. After the agarose gel was centrifuged at 500 × g for 30 s to obtain the gel, it was incubated with 100 μL of the hippocampal homogenate at 4°C overnight. The antigen–antibody binding agarose gels were dissolved in the loading buffer and subsequently centrifuged to extract the supernatant after 5 min of heating at 95°C for Western blot analysis on the second day. We used anti‐IgG secondary antibodies (Vazyme Biotech, 7E710H3) to avoid interference with the IgG chain.

### 5.9 Quantitative Real‐Time PCR

The process of extracting RNA, producing complementary DNA, and conducting qPCR using SYBR Green was carried out following previously published methods (Wu et al. 2024). Briefly, whole RNA was isolated from the hippocampus or cortex via an RNA extraction kit (Vazyme Biotech, R403‐01) with DNase treatment. cDNA reverse transcription was carried out via a reverse transcription kit (Vazyme Biotech, R312–01). Quantitative RT–PCR was performed with SYBR Green PCR Master Mix (Vazyme Biotech, Q321‐02) on a real‐time PCR cycler (LightCycler 480II, Roche LifeScience, Switzerland) with the primers listed in Table 1.

**TABLE 1:** PCR primers employed in the present study.

Gene	Forward primer (5′→3′)	Reverse primer (5′→3′)	Product size (bp)
***Chmp4b***	ATGTCGGTGTTCGGGAAGC	CGGCGCGTTTATTTTTGGTG	193
***Adrb2***	GAGCGACTACAAACCGTCAC	ATTCTTGGTCAGCAGGCTCT	261
***Thap11***	GCTGTGCTTCTTACGCTTCA	CTTGCACTGTGAGGTCGATG	118
***Nr2f6***	CAGATTGATCAGCACCACCG	GTTGTCGATGCCCAGTACAG	267
***Prdm15***	TGGGAGAAAGAGTCGGCATT	TGGTAGGCTGTCAGGTTCTG	149
***Rxrα***	TTTCTGAGCTGCCCCTAGAC	CACGCATCTTAGACACCAGC	210
***Chmp4b promoter Site1***	TGCCACATTGCCCTTTGACCTC	CAAGCCGGGAGTCTGAGTTTTCC	84
***Chmp4b promoter Site2***	TGTTCCAGAGATCATAGAAAATGAGTGC	CGTGATAGAACCCTGGACCTAATTC	83

**TABLE 2:** Antibodies used in the Western blotting analysis and their properties.

Antibody	Specificity	Type	Dilution for WB	Source	Catalogue number
**β‐Actin**	β‐Actin	Poly—	1:5000	SAB	21,338
**CHMP4B**	Total CHMP4B	Mono—	1:1000 for WB	SAB	36,350
**CHMP4B**	Total CHMP4B	Mono—	1:100 for IF	CST	42,466
**CHMP2B**	Total CHMP2B	Mono—	1:1000 for WB	SAB	56,844
**ALIX**	ALIX	Mono—	1:1000 for WB	Proteintech	12,422
**RXRα**	Rxrα	Poly—	1:1000 for WB	Proteintech	21,218
**SYN1**	Synapsin I	Poly—	1:1000 for WB	SAB	41,470
**GRIN1**	NMDAR1	Mono—	1:1000 for WB	SAB	49,488
**GRIN2A**	NMDAR2A	Poly—	1:1000 for WB	SAB	53,091
**GRIN2B**	NMDAR2B	Poly—	1:1000 for WB	SAB	54,739
**PSD95**	PSD95	Poly—	1:1000 for WB	SAB	45,221
**LC3**	Total LC3B	Poly—	1:1000 for WB 1:000 for IF	Abcam	45,394
**β1‐AR**	Beta 1 adrenergic receptor	Poly—	1:1000 for WB	Proteintech	28,323
**β3‐AR**	Beta 3 adrenergic receptor	Poly—	1:1000 for WB	SAB	55,556
**β2‐AR**	Beta 2 adrenergic receptor	Mono—	1:1000 for WB 1:100 for IF	Abcam	182,136
**SQSTM1**	SQSTM1	Poly—	1:1000 for WB 1:100 for IF	CST	23,214
**Tau5**	Tau5	Mono—	1:1000 for WB 1:200 for IF	SAB	21,570
**Tau5**	Tau5	Mono—	1:100 for WB 1:50 for IF	Santa cruz	Sc‐58,860
**pT231**	Phospho‐Tau (Thr231)	Mono—	1:1000 for WB	SAB	21,099
**pS396**	Phospho‐Tau (Thr396)	Mono—	1:1000 for WB	SAB	11,102
**pS404**	Phospho‐Tau (Thr404)	Mono—	1:1000 for WB	SAB	Bs‐ 2392R
**pSβ2‐AR**	Phospho‐β2‐AR (Ser355/s356)	Poly—	1:1000 for WB	SAB	14,073–1
**Neuron**	Neuron	Mono—	1:100 for IF	Abcam	104,224
**HT7**	Tau Antibody	Mono—	1:1000 for WB	Thermo Fisher	MN1000

Abbreviations: mono, monoclonal; poly, polyclonal.

**TABLE 3:** Information of human brain sections.

	Type	Dementia degree	Age	Gender	PMI (h)	Cause of death	Braak Stage
1	HC	−	25	Female	12	Fall to death	−
2	HC	−	62	Female	84	Carotid artery rupture	−
3	HC	−	48	Male	96	Organophosphorus	−
4	HC	−	52	Male	120	Fall to death	−
5	AD	+++	74	Male	12	Acute myocardial ischemia	III
6	AD	++	53	Male	48	Hypertension	III
**7**	AD	++	62	Female	72	Septicopyemia	II
8	AD	+	58	Male	144	Hemorrhagic shock	I

Abbreviations: AD, Alzheimer's disease; HC, health control; PMI, post mortem interval.

### 5.10 Immunofluorescence

The brain hemispheres were immersed in paraformaldehyde fixative for more than 2 weeks. After dehydration, the mouse brains were sliced into slices of approximately 14 μm using a freezing slicer. The brain slices were permeabilized for 30 min and then blocked for 1 h, after which they were incubated according to the antibody instructions. The slices were subsequently incubated at 37°C for 1 h with conjugated secondary antibodies, either Alexa Fluor 488 (Jackson ImmunoResearch, 711–545‐152) or Alexa Fluor 594 antibodies (Jackson ImmunoResearch, 715–585‐150), along with a Multiple Staining Fluorescence Kit (Fcmacs Biotech, FMS‐Mihc001‐20 T). Images were acquired via a confocal fluorescence microscope (Axio Imager Z2, Carl Zeiss, Germany).

### 5.11 Cell Culture and Transfection

Mouse neuroblastoma Neuro2a cells (N2a) (Probiotic, CL0168, China) were cultured in DMEM (Cytiva, SH30243.01, USA) supplemented with 10% fetal bovine serum (Bioexplorer, BS1614‐109, USA) and 1 × penicillin–streptomycin solution (Biosharp, BL505A, China) in a humidified incubator containing 5% CO_2_. The cells were cultured on well plates to approximately 70% confluence and then transfected with plasmids encoding untagged full‐length human Tau (hTau) and 3xFlag‐Tau using Lipofectamine RNAiMAX (Invitrogen). The post‐transfection cells were treated with the β2‐AR agonist Teb (MedChemExpress, HY‐B0802A) at 10 μM for 1 h for further protein extraction and immunofluorescence experiments. The CHMP4B and RXRα overexpression plasmids were generated by YouBio (Hunan, China). The siRNA targeting β2‐AR was created and produced by RiboBio Technology (Guangzhou, China). The siRNAs targeting CHMP4B and RXRα were produced by GenePharma (Shanghai, China). The sequence of si‐*Chmp4b* was as follows: AGAAAGAAGAGGAGGACGAtt, as previously designed (Mierzwa et al. 2017), and the sequence of *Rxrα* was AGACCUACGUGGAGGCAAA, as previously designed (Ma et al. 2018).

Autophagic flow experiments were performed via the transfection of a tandem monomeric Cherry‐GFP‐tagged LC3 (mRFP‐GFP‐LC3) plasmid, as well as plasmids encoding untagged full‐length human Tau (hTau) and 3 × Flag‐Tau, all of which were donated by Prof. Jianzhi Wang and Prof. Gongping Liu (Tongji Medical School, Wuhan, China).

### 5.12 Transmission Electron Microscopy

Hippocampal tissues were isolated, rapidly placed in 2.5% glutaraldehyde for fixation for more than 4 h, and then sent to the Shiyanjia Laboratory (www.shiyanjia.com) for sectioning. Following a 15–20 min dehydration period using a graded sequence of ethanol (30%, 50%, 70%, 80%, 90%, 95%, and 100%), the sample was placed in absolute acetone for 30 min. This step was followed by a 3 h incubation in a 1:3 mixture of absolute acetone and the final resin, after which the samples were left overnight in the Spurr resin. Finally, the sample was heated to 70°C for more than 9 h within a tube filled with Spurr resin to facilitate ultrathin sectioning and embedding. The sections were then stained for 5 to 10 min and examined via a Hitachi Model H‐7650 transmission electron microscope. For quantifying synaptic ultrastructures, we followed a published study that analyzed synapse intensity, postsynaptic density (PSD) thickness, and synaptic cleft width (Restivo et al. 2009). To assess autophagosome numbers and synapses, three consecutive slices from the CA1 region of each mouse were selected for quantitative analysis.

### 5.13 Golgi Staining

The mice were anesthetized with 20% ouabain, and their brains were rapidly isolated. The surface blood was then rinsed with distilled water, and the samples were stained with a Rapid Golgi Stain kit (FD Neuro Technologies PK40, USA). In brief, the brains were removed, the blood on the surface was rinsed with distilled water, and then immersed in impregnation solution, prepared by mixing equal volumes of solutions A and B, for 2 weeks. After incubation with solution C, the brains were sliced into approximately 100 μm sections using a freezing slicer. The sections were stained with D + E solution for 10 min, dehydrated in alcohol, and then imaged under a light field. Sholl analysis and assessment of dendritic spines were conducted via ImageJ or Fiji software following previously established protocols (Liu et al. 2015; Tang et al. 2019).

### 5.14 ChIP

The experimental procedure adhered to the guidelines provided by the ChIP assay kit (Beyotime, P2078, China). In brief, the hippocampal tissue from the mice was crosslinked with formaldehyde for 15 min at room temperature. This mixture was subsequently mixed with glycine for 10 min and then rinsed twice with cold PBS. The tissues were ultrasonicated on ice with an ultrasound device (Scientz, IID, China), and the supernatant was diluted with ChIP buffer. For the minimization of nonspecific binding, agarose CHMP4B A + G/salmon sperm DNA was added. After centrifugation, the samples were incubated with antibodies at 4°C following the antibody dilution instructions. On the second day, three different gradients of salt immunocomplex wash buffer were used, and the samples were washed once with TE buffer (50 mM Tris–HCl and 10 mM EDTA). DNA purification was then carried out via a kit (Beyotime, D0033, China). Afterward, qPCR was performed on the DNA pellets.

### 5.15 Dual Luciferase Activity Assay

The RXRα binding sequence within the promoter region of the CHMP4B gene was synthesized and inserted into the pDualuc reporter vector by YouBio (Hunan, China). Following cotransient transfection of transcription factors and dual‐luciferase vectors into HEK293T (CRL‐3216, USA) cells, a dual‐luciferase assay was subsequently performed with a kit (Beyotime, RG088S, China) according to the experimental procedure instructions. The fluorescence intensity was assessed with a microplate reader (Synergy H4, USA). Every experiment was carried out at least three times. The supplemental table includes a list of the combination sequences.

### 5.16 Transcriptomic

CHMP4B mRNA expression data from whole‐brain tissues in the GSE5281 and GSE15222 datasets were obtained from the AlzData differential expression platform (http://www.alzdata.org/Normalized_differential1.php), and differential expression was determined based on a significance threshold of *p*‐value < 0.05. The transcriptomic dataset GSE110298, derived from the Gene Expression Omnibus (GEO) database (https://www.ncbi.nlm.nih.gov/geo/), profiles exercise‐associated gene expression changes in AD. The mRNA expression data were preprocessed, and batch effects were removed using the “sva” package in R (R version 4.2.1). Differential expression analysis was performed using the “limma” package, and genes with a *p*‐value < 0.05 and an absolute log_2_ fold change (|log2FC|) > 0.263 were defined as differentially expressed genes (DEGs). Subsequently, Gene Ontology biological process (GO‐BP) and Kyoto Encyclopedia of Genes and Genomes (KEGG) pathway enrichment analyses were conducted based on DEGs using an online platform (https://bioinformatics.com.cn/), with a significance threshold set at *p*‐value < 0.05. To further explore the potential molecular interactions among DEGs, a protein–protein interaction (PPI) network analysis was performed using the STRING database (https://cn.string‐db.org/), and the network was visualized with Cytoscape software (version 3.9.1).

Physical activity levels were monitored using wrist‐worn actigraphy over 10 consecutive days in dataset GSE110298. Based on total daily counts converted into metabolic equivalents (METs), individuals performing > 26 MET‐hours/week (equivalent to > 5.0 × 10^5^ counts/day or ~100 min/day of moderate‐to‐high intensity activity) were defined as the high physical activity group. Those below this threshold were classified as the low physical activity group. This cutoff was chosen based on prior evidence linking ≥ 26 MET‐hours/week with optimal cognitive outcomes (Berchtold et al. 2019). All statistical methods applied were derived from the experimental procedures described in the GSE110298 dataset (Berchtold et al. 2019).

### 5.17 Extraction of Soluble and Insoluble MAPT

The isolation of soluble and insoluble MAPT fractions from mouse hippocampal tissue was performed as previously described (Feng et al. 2020). In brief, the hippocampal tissue was homogenized in supplemented lysis buffer and then centrifuged at 14,000 × g for 15 min at 4°C, after which the supernatant was collected as the soluble MAPT fraction. The pellet underwent a second round of homogenization and centrifugation under the same conditions. The pellet was subsequently resuspended and stirred overnight in a 1:1 (w: v) solution of 70% formic acid at 4°C. After this incubation, the suspension was centrifuged at 18,000 × g for 20 min at 4°C to collect the supernatant. Following the evaporation of formic acid, the resulting precipitates were resuspended in loading buffer in preparation for immunoblotting.

### 5.18 Statistical Analysis

Data were analyzed using GraphPad Prism software 9 (GraphPad Software Inc., La Jolla, CA, USA). Statistical analyses included the unpaired t‐test, one‐way analysis of variance (ANOVA), and two‐way ANOVA followed by Bonferroni's post hoc test. The data were expressed as mean ± SEM, and *p*‐values < 0.05 were considered to be significant.

## Author Contributions

Experimental design: S.‐G.B., H.Y., and G.‐S.C. Experimental methods: S.‐G.B., H.Y., Y.‐M.M., J.‐J.W., T.‐L.G., and J.G. Data analysis: S.‐G.B., F.‐Z.W., L.Y., J.C., Z.‐C.L., M.‐T.S., and Y.‐J.N. Manuscript writing: S.‐G.B. and G.‐S.C.

## Disclosure

The authors have nothing to report.

## Conflicts of Interest

The authors declare no conflicts of interest.

## Supporting information


**Data S1:** acel70184‐sup‐0001‐DataS1.docx.

## Data Availability

Data sharing is not applicable to this article as no new data were created or analyzed in this study.
