# Supplementary material for: Aerobic Exercise Attenuates Autophagy‐Lysosomal Flux Deficits via β2‐AR‐Mediated ESCRT‐III Subunit CHMP4B in Mice With Human MAPT P301L
Source: Aging Cell. 2025 Jul 26;24(10):e70184. doi: 10.1111/acel.70184 (PMC12507411; doi:10.1111/acel.70184)
Supplement: Supplementary file 1 — Data S1: acel70184‐sup‐0001‐DataS1.docx. [file ACEL-24-e70184-s001.docx]

**Supplementary Materials for**

**Aerobic exercise attenuates autophagy-lysosomal flux deficits via β2-AR-mediated ESCRT-III subunit CHMP4B in mice with human MAPT P301L**

Shu-guang Bi *et al.*

*Corresponding author: Gao-shang Chai, Email: [chaigaoshang@jiangnan.edu.cn](mailto:chaigaoshang@jiangnan.edu.cn)

**Supplementary Figures legends**


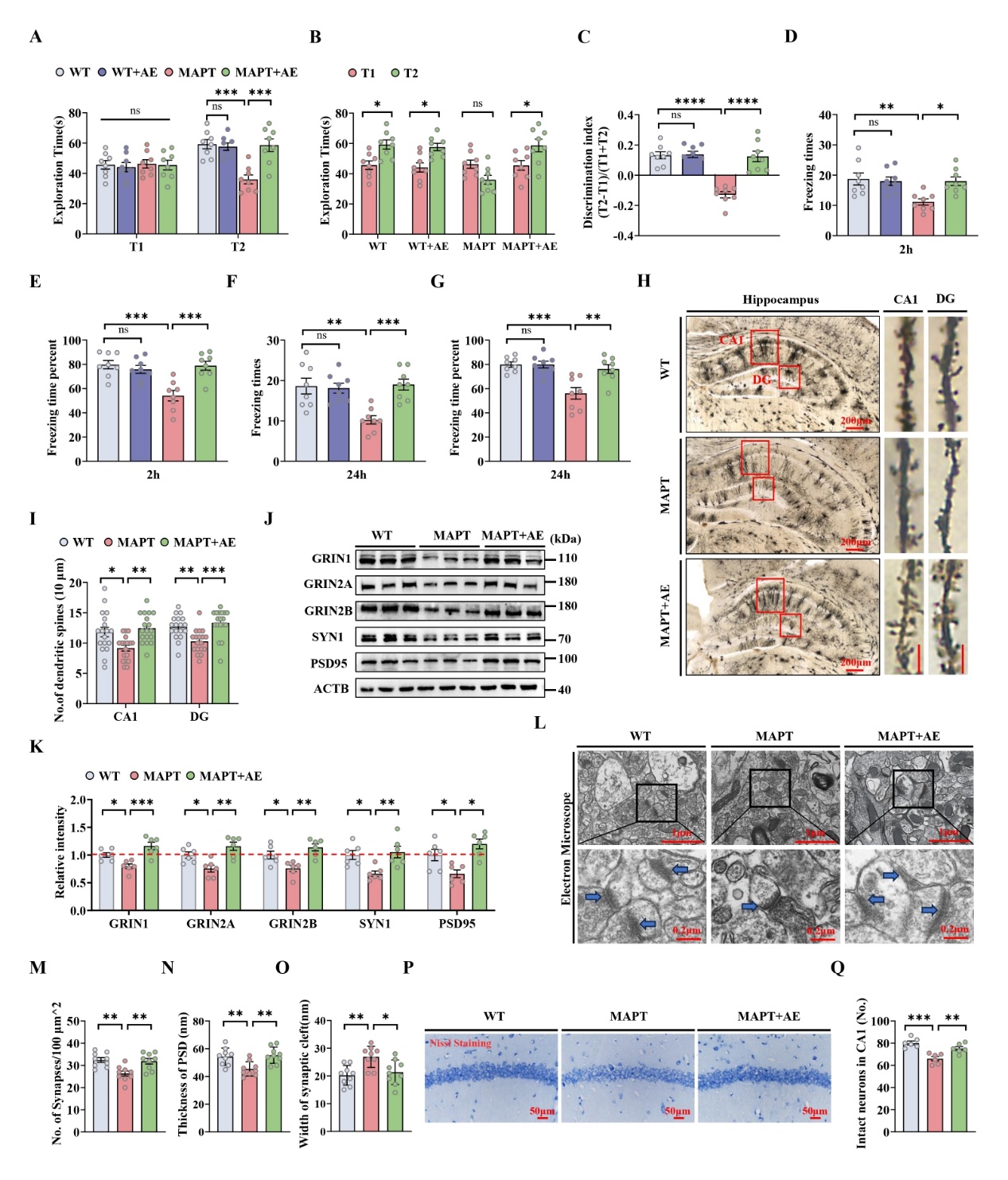
**Figure S1. AE ameliorates cognitive deficits and MAPT pathology in MAPT P301L mice.** (**A-C**) Effect of AE on the Novel Object Recognition (NOR) test of WT, WT+AE, MAPT and MAPT+AE mice. n = 8 mice per group test (**A**) The time mice spent exploring old and novel objects among 4 groups in the NOR test (T1: Time to identify old objects; T2: Time to identify novel objects). (**B**) Time to identify old and novel objects within each group. (**C**) The discrimination index of mice was calculated by (T2-T1):(T1+T2). (**D-G**) Effect of AE on the foot shock-induced Fear Conditioning Test (FCT). (**D and E**) Freezing times and freezing time percent in test 2 h later. (**F and G**) Freezing times and freezing time percent in the test after 24 h. (**H**) Representative images of hippocampal CA1 and DG neurons with Golgi staining (scale bar: 5 μm). (**I**) Quantification of the number of dendritic spines in hippocampal CA1 and DG regions (n = 18 neurons from 3 mice per group were analyzed). (**J and K**) The protein levels of GRIN1, GRIN2A, GRIN2B, SYN1 and PDS95 in the hippocampus were detected using western blotting and quantitatively analyzed (n = 6 mice per group). (**L**) Electron microscopy analysis of ultrastructure of synapses in the hippocampus. (**M-O**) Quantitative analysis of synaptic density, the thickness of PSD and the width of the synaptic cleft (n = 9 images from 3 mice per group were analyzed). (**P and Q**) Nissl staining and quantification of intact neurons in the hippocampal CA1 region of mice. Neurons with visible nuclei, prominent nucleoli, and Nissl substance-positive cytoplasm were defined as intact neurons. (n = 6 mice per group). * *p* < 0.05; ** *p* < 0.01; *** *p* < 0.001; **** *p* < 0.0001. The data were presented as the means ± SEMs. Two-way ANOVA with Bonferroni post hoc test was used for (**B**), and one-way ANOVA with Bonferroni post hoc test for the other data.


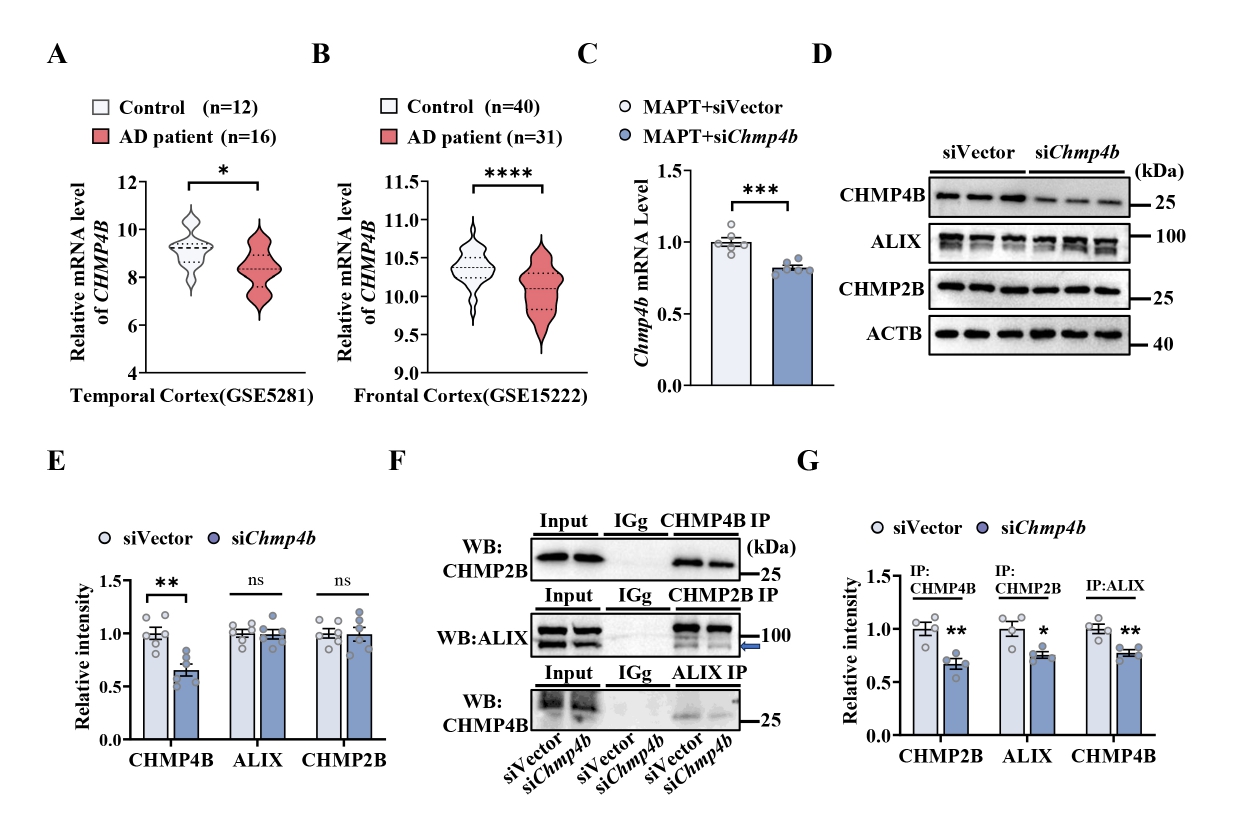
**Figure S2. CHMP4B deficits underlie MAPT-induced autophagic repression by disrupting ESCRT-III assembly.** (**A and B**) Transcriptional down-regulation of CHMP4B in temporal cortex tissue from AD patients in the GSE5281 (control, n = 12; AD patient, n = 16) and in frontal cortex tissue from AD patients in the GSE15222 (control, n =40; AD patient, n =31). (**C**) The mRNA level of *Chmp4b* in N2a cells treated with siVector and si*Chmp4b*. (**D and E**) The levels of CHMP4B, ALIX and CHMP2B in N2a cells treated with siVector and si*Chmp4b* (n = 6 independent experiments for each group). (**F and G**) Co-immunoprecipitation (Co-IP) assay revealed the interaction among CHMP4B, CHMP2B and ALIX in N2a cells treated with si*Chmp4b* (n = 4 independent experiments for each group). * *p* < 0.05; ** *p* < 0.01; *** *p* < 0.001; **** *p* < 0.0001. The data were presented as the means ± SEMs. Unpaired t-test was used to analyze the data.


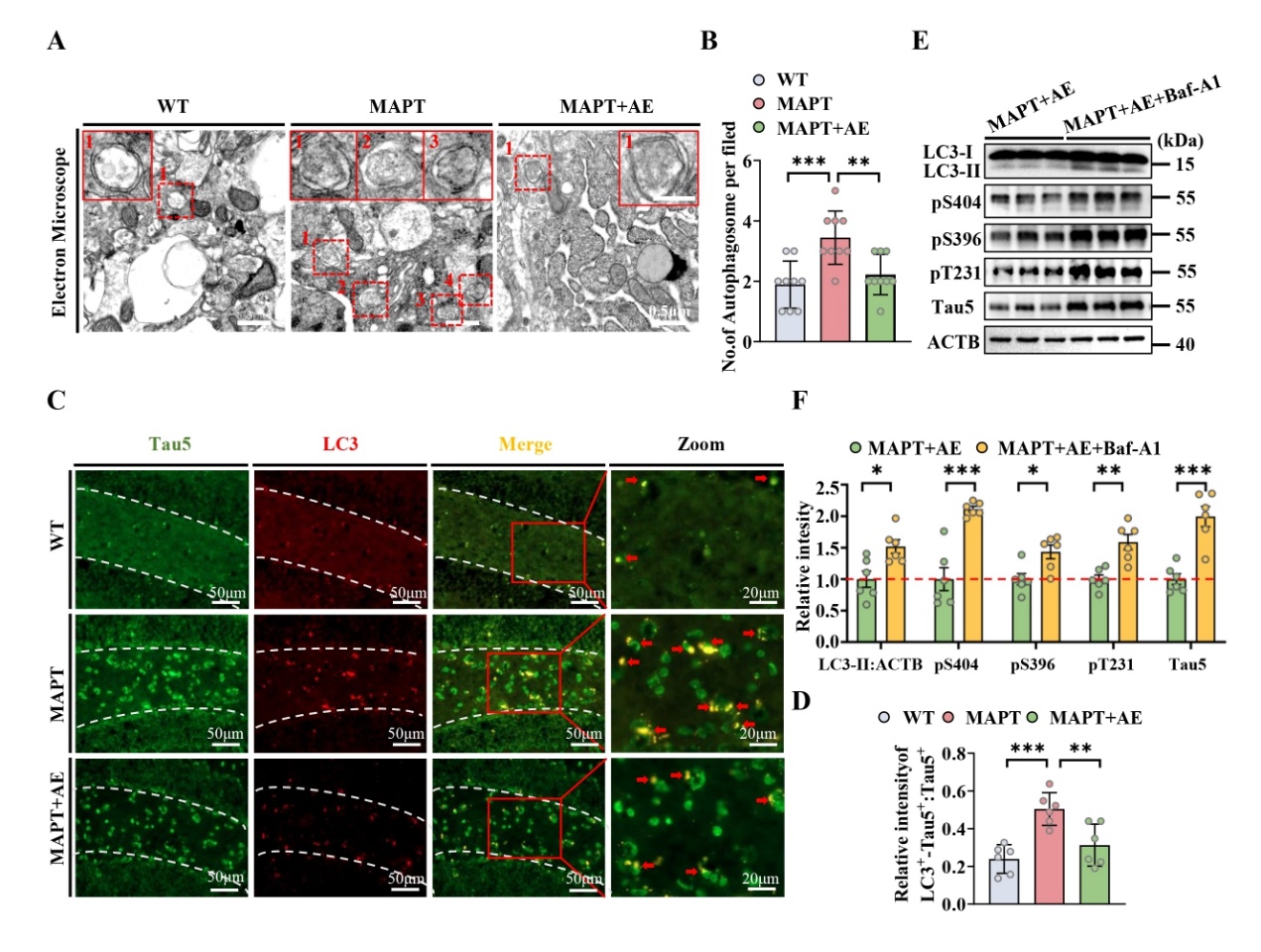
**Figure S3. AE promotes autophagic progression by increasing autophagosome degradation in MAPT P301L mice.** (**A**) Electron microscopy analysis of autophagosomes. Arrows indicated the autophagosomes (scale bar:0.5 μm). (**B**) Quantification of autophagosomes (n = 9 images from 3 mice per group were analyzed). (**C**) Representative images of LC3 and Tau5 immunofluorescence co-labeling in the hippocampus DG region of the mice. (**D**) Quantification of the intensity of the ratio of LC3^+^Tau5^+^: Tau5^+^ (n = 6 mice per group). (**E and F**) Representative images and quantification of LC3-II, soluble Tau of (pS396, pS404, pT231 and total Tau5) in the hippocampus of AE mice after Baf-A1 treatment (n = 6 mice per group). * *p* < 0.05; ** *p* < 0.01; *** *p* < 0.001; **** *p* < 0.0001. The data were presented as the means ± SEMs. One-way ANOVA with Bonferroni post-hoc test was used to analyze the data in (**B, D**), and unpaired t-test was used to analyze the data in (**F**).


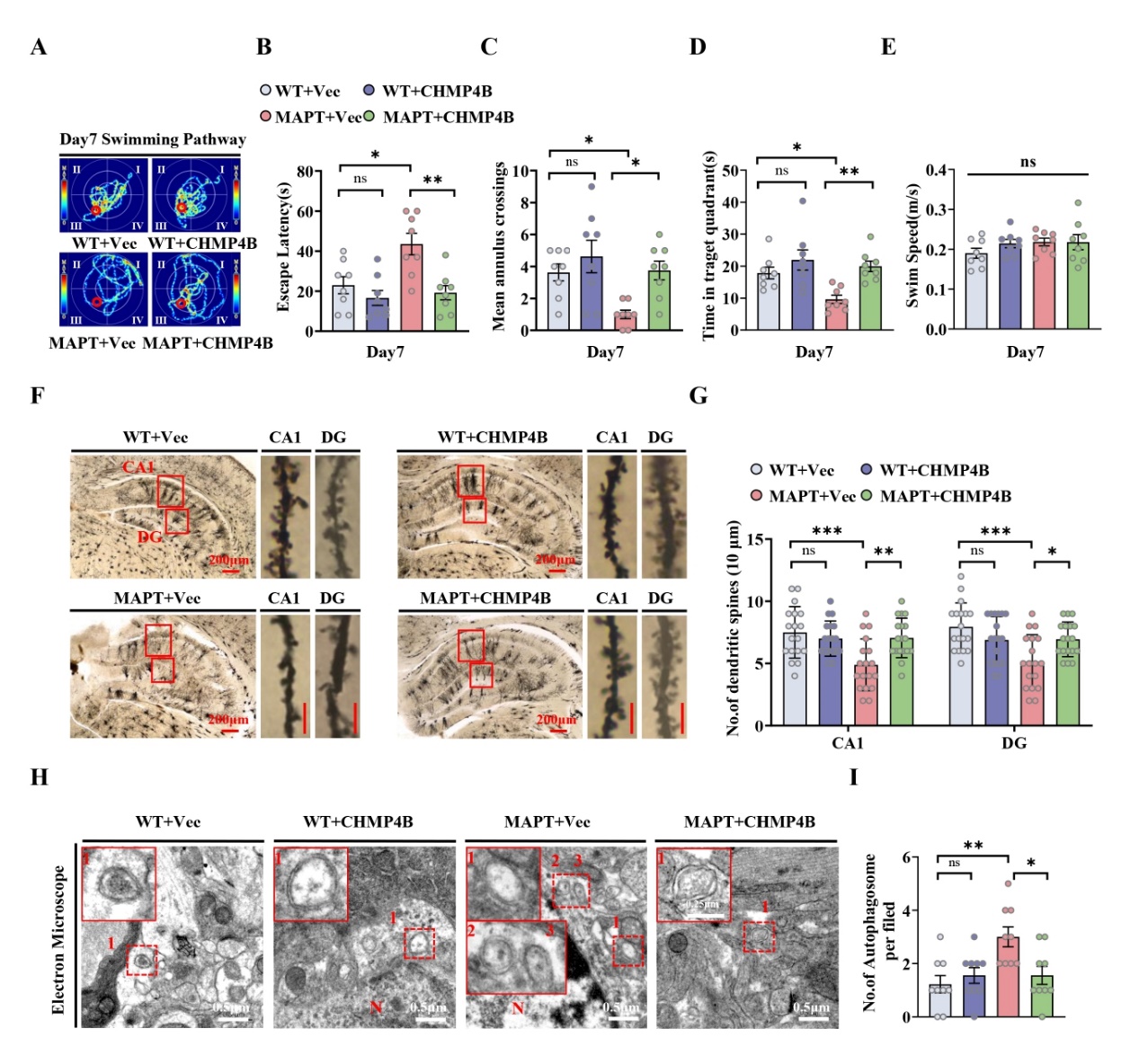
**Figure S4. Upregulating CHMP4B ameliorates cognitive deficits, synaptic plasticity in MAPT P301L mice.** (**A**) The swimming pathway traveled to locate the platform on day 7 in WT+Vec, WT+CHMP4B, MAPT+Vec and MAPT+CHMP4B mice. (**B**) Escape latency, (**C**) number of crossings of the hidden platform area, (**D**) the time in target quadrants and (**E**) swimming speed on day 7. (**F and G**) Quantification of the number of dendritic spines in hippocampal CA1 and DG regions. (n=18 neurons from 3 mice per group were analyzed). (**H**) Electron microscopy analysis of autophagosomes. Arrows indicate the autophagosomes (scale bar:0.5 μm). (**I**) Quantification of autophagosomes (n = 9 images from 3 mice per group were analyzed). * *p* < 0.05; ** *p* < 0.01; *** *p* < 0.001; **** *p* < 0.0001. The data were presented as the means ± SEMs. One-way ANOVA with Bonferroni post hoc test was used to analyze the data.


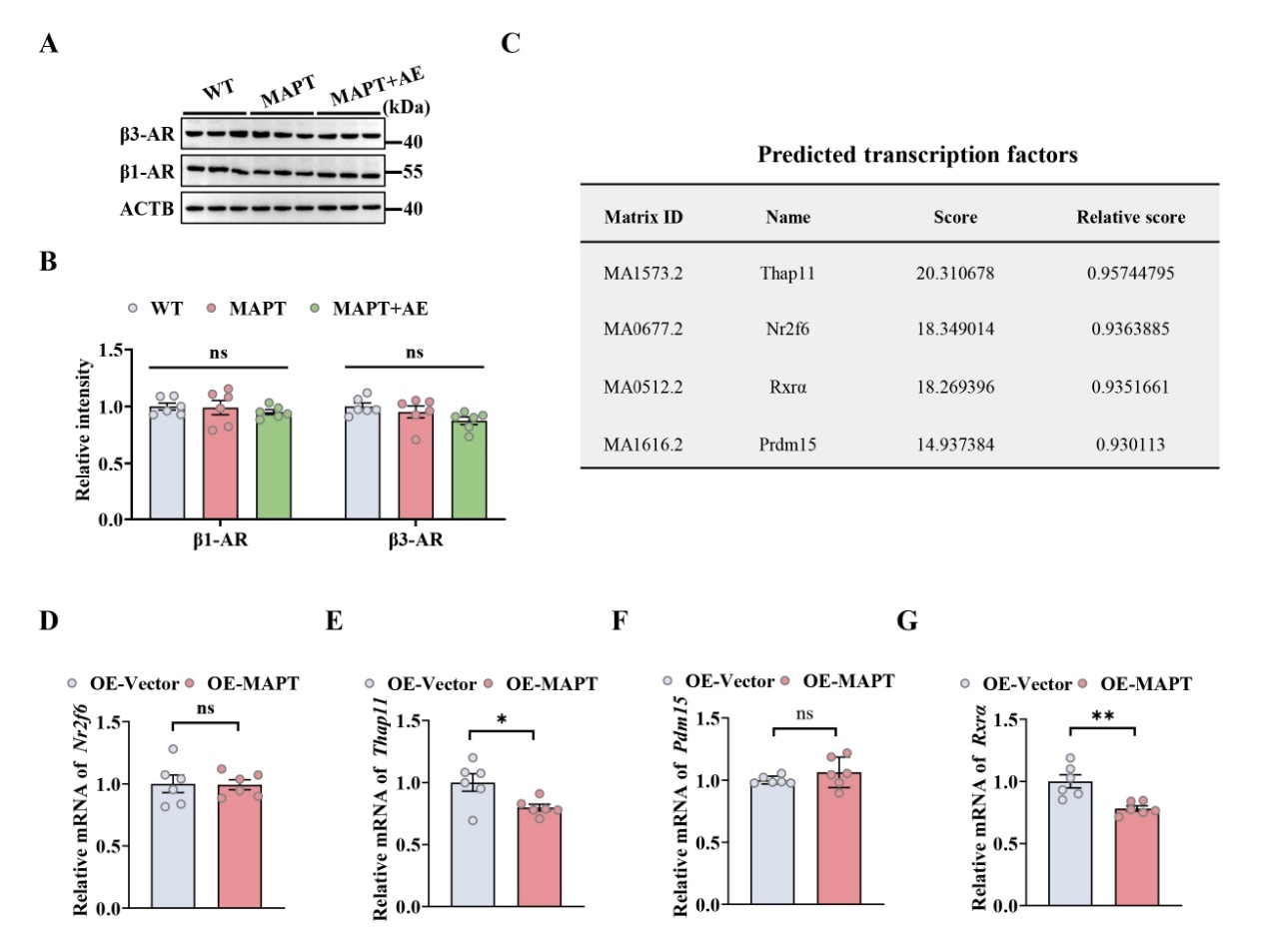
**Figure S5. Overexpression of MAPT promotes CHMP4B partial transcription factor expression in N2a cells.** (**A and B**) The protein levels of β1-AR and β3-AR in the hippocampus of WT, MAPT and AE-treated MAPT mice (n = 6 mice per group). (**C**) Transcription factor prediction of CHMP4B was performed by NCBI and Jaspar. The list showed the top 4 candidate genes overall. (**D-G**) The mRNA levels of the CHMP4B transcription factors of *Thap11*, *Nr2f6*, *Prdm15* and *Rxrα* in N2a cells with overexpression of MAPT and Vector (n = 6 independent experiments for each group). * *p* < 0.05; ** *p* < 0.01; *** *p* < 0.001; **** *p* < 0.0001. The data were presented as the means ± SEMs. One-way ANOVA with Bonferroni post hoc test was used to analyze the data in (**B**), and unpaired t-test was used to analyze the other data.


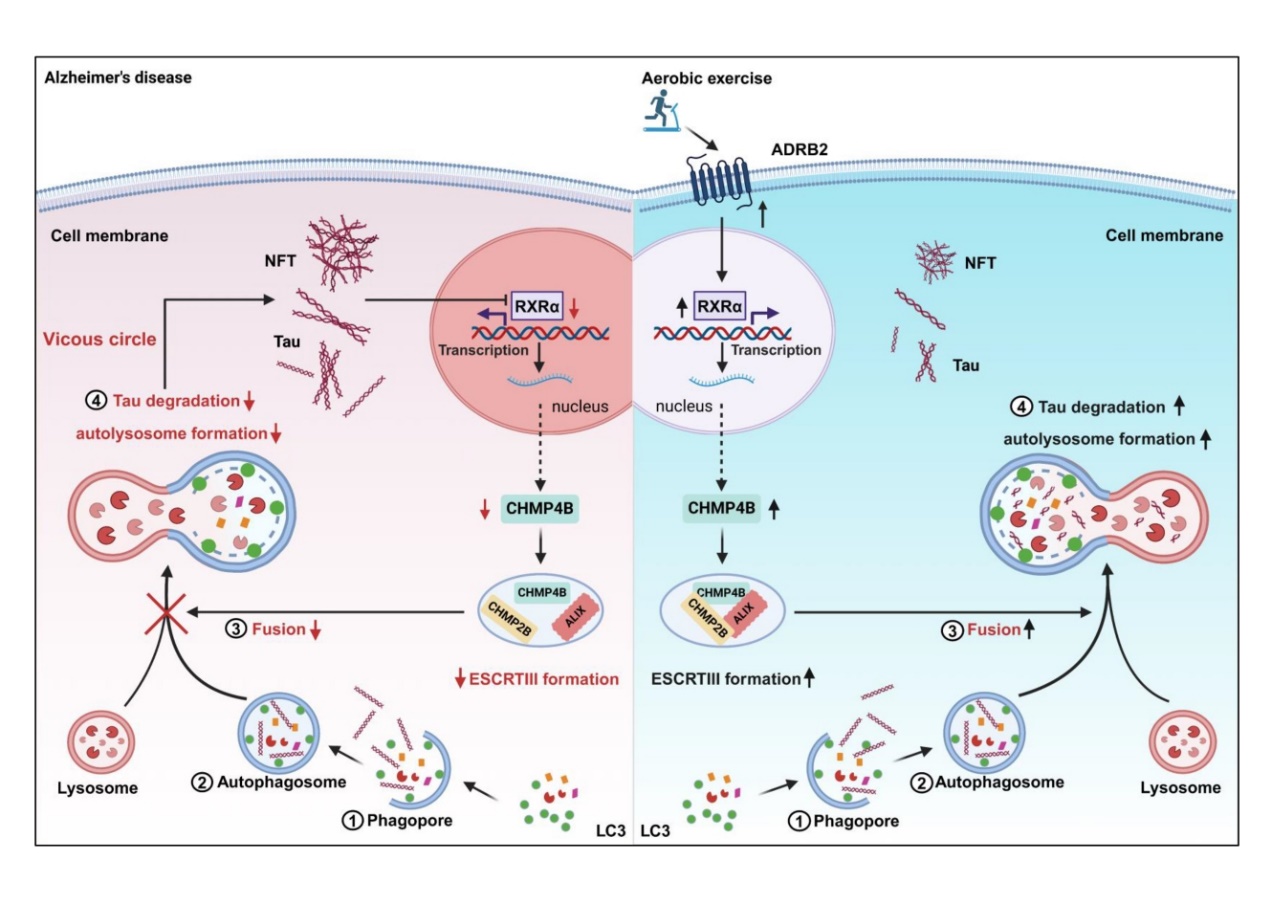
**Figure S6. Proposed working model for aerobic exercise attenuates autophagosome-lysosome fusion deficits via β2-AR-RXRα-CHMP4B-ESCRT-III pathway signaling in Alzheimer disease mice.**
